# Resveratrol/Hydrazone Hybrids: Synthesis and Chemopreventive Activity against Colorectal Cancer Cells

**DOI:** 10.3390/pharmaceutics14112278

**Published:** 2022-10-24

**Authors:** Wilson Castrillón-López, Angie Herrera-Ramírez, Gustavo Moreno-Quintero, Juan Carlos Coa, Tonny W. Naranjo, Wilson Cardona-Galeano

**Affiliations:** 1 Chemistry of Colombian Plants Group, Institute of Chemistry, Faculty of Exact and Natural Sciences, University of Antioquia, Medellín 050010, Colombia; 2 Medical and Experimental Mycology Group, Corporación para Investigaciones Biológicas, Medellín 050034, Colombia; 3 School of Health Sciences, Pontificial Bolivarian University, Medellín 050034, Colombia

**Keywords:** resveratrol, hydrazone, hybrid compounds, colorectal cancer, cytotoxicity, antiproliferative activity, mitochondrial depolarization, apoptosis

## Abstract

A series of resveratrol/hydrazone hybrids were obtained and elucidated by spectroscopic analysis. All compounds were evaluated against colorectal cancer cells (SW480 and Sw620) and nonmalignant cell lines (HaCaT and CHO-K1) to establish the selectivity index. Among the hybrids evaluated, compounds **6e** and **7** displayed the highest cytotoxic activity with IC_50_ values of = 6.5 ± 1.9 µM and 19.0 ± 1.4 µM, respectively, on SW480 cells. In addition, hybrid **7** also exhibited activity on SW620 cells with an IC_50_ value of 38.41 ± 3.3 µM. Both compounds were even more toxic against these malignant cells in comparison to the nonmalignant ones, as evidenced by higher selectivity indices 48 h after treatment. These compounds displayed better activity and selectivity than parental compounds (PIH and Resveratrol) and the reference drug (5-FU). In addition, it was observed that both compounds caused antiproliferative activity probably exerted by cell cycle arrest at the G2/M or G0/G1 phases, with the formation of cells in the subG0/G1 phase. Furthermore, it was noticed that compound **7** induced mitochondrial depolarization in SW480 cells and positive staining for propidium iodide in both cancer cell lines, suggesting cell membrane damage involving either apoptosis or other processes of death.

## 1. Introduction

Colorectal cancer (CRC) is the third most diagnosed malignancy and the fourth leading cause of cancer death worldwide [1]. This pathology presents a great geographical distribution, and the patterns are very similar among men and women, being the third most common cancer in men and the second one in women [2]. These statistics are surprisingly high despite the fact that CRC is considered one of the most preventable cancers through modifications in lifestyles [3,4]. Current chemotherapy for CRC is mainly based on 5-fluorouracil, which is used in combination with oxaliplatin, irinotecan, leucovorin, or folic acid in different therapeutic regimes. Although these treatments are effective, they induce a high grade of toxicity, including neurological and gastrointestinal disorders, myelosuppression, anemia, and neutropenia, among others [5,6,7,8]. Considering the disadvantages of conventional chemotherapy, there is an urgent need for developing novel approaches for the treatment of colorectal cancer.

**Figure 1:**
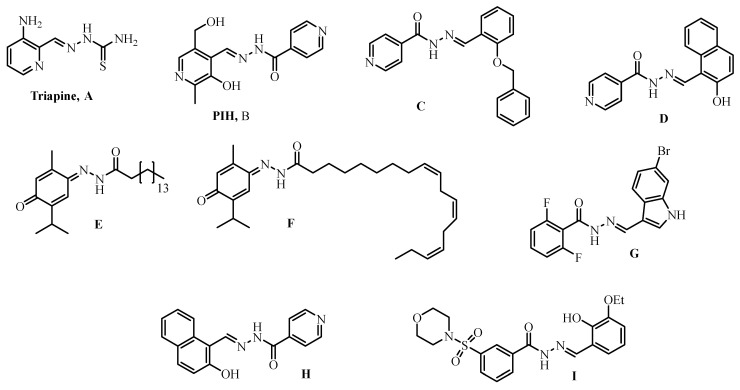
Hydrazones with anti-tumoral activity.

Chemoprevention has emerged as a relatively new field in the search for new therapeutic alternatives. This strategy is based on the use of natural, synthetic, or biological alternatives to reverse, suppress, or either prevent any of the steps in carcinogenesis (initiation, promotion, or progression) [9,10]. Thereon, the use of hydrazones has been evaluated. They constitute an important type of compound with a plethora of pharmacological activities [11,12,13], for this reason, different compounds containing the hydrazone moiety are currently being used in clinical trials, including pyridoxal isonicotinoyl hydrazone (PIH, **A**) and triapine **B** (Figure 1) [14]. These compounds are classified as chelating agents used in the treatment of iron overload diseases. Besides, considering the associations between iron and cancer, they have also been investigated in different models due to their ability to inhibit the growth of aggressive tumors in vitro and in vivo [15,16,17]. Several hydrazones have been explored because of their anticancer potential. Among them, compounds **C** and **D** displayed antiproliferative activity with IC_50_ values of 3.1 and 0.29 µM, respectively, against HCT 116 cell line. These results were even better than the known values for the drug 5-fluorouracil (IC_50_ = 5 µM) [18]. On the other hand, in a different investigation it was shown that hydrazones **E** and **F** induced a cytostatic effect in p53-competent HCT116 cells, mediated by up-regulation of p21cip1/waf1 and a down-regulation of cyclin E, besides, the authors reported an association with cell cycle arrest at the S/G2 phase [19]. Moreover, acylhydrazone **G** exhibited toxicity when evaluated in a panel of colon cancer cells (HCT-116, DLD-1, and SW620), without damaging nonmalignant L929 fibroblasts. Furthermore, this compound triggered programmed cell death (early and late apoptosis) through cell cycle arrest in the G2/M phase and caspase-9/3 cleavage in HCT-116 colon cancer cells [20]. In addition, compound **H** (NIH) showed greater antiproliferative activity than PIH, displaying high chelation efficacy which plays an essential role in its cytotoxic activity [21]. Finally, compound **I** showed strong antiproliferative activity on esophageal carcinoma cells (EC9706 and EC109) with IC_50_ values of 1.09 ± 0.03 and 2.79 ± 0.45 µM, respectively. This hydrazone also induced apoptosis and cell cycle arrest at G0/G1 phase in both cell lines [22]. All these structures are illustrated in Figure 1.

**Figure 2:**
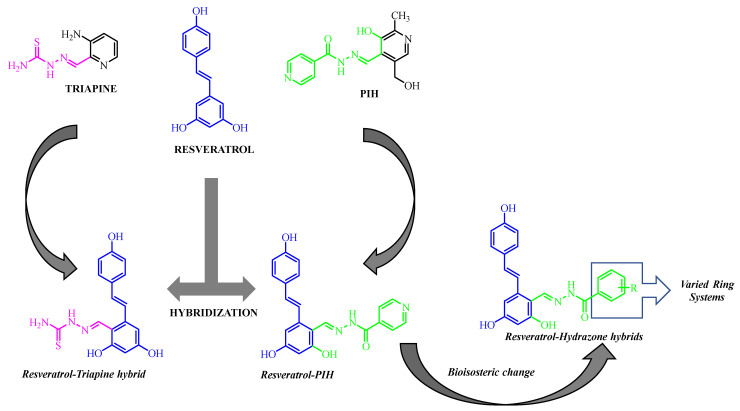
Design of resveratrol/hydrazone hybrids as anticancer agents.

The bioactive natural product resveratrol (Figure 2), has received great attention in drug discovery since it has displayed several biological activities including antitumor [23,24]. This stilbene has demonstrated effectiveness in all the stages of carcinogenesis (initiation, promotion, and progression), by modulating signal transduction pathways that control cell division and growth, apoptosis, inflammation, angiogenesis, and metastasis [25,26,27,28,29,30,31]. Despite its effectiveness, the clinical use of resveratrol is limited because of its low bioavailability and stability [32,33].

Considering the limitations of conventional chemotherapy and the individual molecules, the use of molecular hybridization has emerged as a promising strategy in medicinal chemistry. This strategy is used in the search for new therapeutic alternatives and it is based on the linking of two different pharmacophores to create a single molecule that could exhibit a dual mode of action but does not necessarily on the same biological target [34,35,36,37]. Based on this strategy and the high potential of resveratrol and hydrazones as anticancer agents, we decided to synthesize a series of resveratrol/hydrazone hybrids (Figure 2) and tested their biological activity using two human colon cancer cells (SW480 and SW620) establishing an approach towards the possible chemopreventive potential of the synthesized molecules.

## 2. Materials and Methods

### 2.1. Chemical Synthesis

#### 2.1.1. General Remarks

Microwave reactions were carried out in a CEM Discover microwave reactor in sealed vessels (monowave, maximum power 300 W, temperature control by IR sensor, and fixed temperature). ^1^H and ^13^C NMR spectra were recorded on a Varian instrument operating at 300 and 75 MHz, respectively. The signals of the deuterated solvent (DMSO-D6) were used as a reference. Chemical shifts (δ) are expressed in ppm with the solvent peak as a reference and TMS as an internal standard; coupling constants (J) are given in Hertz (Hz). HRMS was obtained using a Bruker Impact II UHR-Q-TOF mass spectrometry (Bruker Daltonik GmbH, Bremen, Germany) in positive mode. Silica gel 60 (0.063–0.200 mesh, Merck, Whitehouse Station, NJ, USA) was used for column chromatography, and precoated silica gel plates (Merck 60 F254 0.2 mm) were used for thin layer chromatography (TLC). Monitoring of the reaction progress and product purification was carried out by TLC.

#### 2.1.2. Synthetic Procedure for Benzohydrazides (**3**)

Hydrazine monohydrate (3 mL of an 80% solution) was added to a solution of 2 (1 mmol) in ethanol (15 mL). The reaction mixture was submitted to microwave radiation and maintained under reflux for 1 h. Then, the crude reaction was concentrated on a rotatory evaporator, and the residue was purified by column chromatography over silica gel to obtain the title compound **3** in 60–90% yield.

#### 2.1.3. Synthetic Procedure for Hydrazone

A benzohydrazides 3 (1 mmol) solution in methanol (5 mL) was sonicated for 2 min and resveratrol-aldehyde 5 (1 mmol) and acetic acid (1 mL) were added dropwise. Then, the reaction mixture was sonicated for 30 min at 40 °C. The product was filtered, sequentially washed with water (20 mL) and ethyl ether (5 mL), dried in vacuo, and recrystallized from ethanol, affording the corresponding hydrazones in yields ranging from 55–70%. The ^1^H, ^13^C NMR, and MS spectra (Supplementary S1) and HPLC analysis (Supplementary S2) of all hybrids can be found in the Supplementary Materials.

N′-((*E*)-2,4-dihydroxy-6-((*E*)-4-hydroxystyryl)benzylidene)-3-methoxybenzohydrazide (**6a**): Yield 55%; Yellow solid. Mp 248–250 °C. ^1^H NMR (300 MHz, DMSO-d6) δ 12.40 (s, OH), 11.93 (s, OH), 8.98 (s, -CH=N-), 7.55–7.40 (m, 5H), 7.32 (d, *J* = 16.0 Hz, 1H), 7.21–7.14 (m, 1H), 6.93 (d, *J* = 16.0 Hz, 1H), 6.80 (d, *J* = 8.5 Hz, 2H), 6.57 (d, *J* = 2.2 Hz, 1H), 6.26 (d, *J* = 2.1 Hz, 1H), 3.83 (s, 3H). ^13^C NMR (75 MHz, DMSO-d6) δ 162.48 (C=O), 161.23 (Ar-O), 160.90 (Ar-O), 159.73 (Ar-O), 158.16 (Ar-O), 148.61 (C=N), 141.00, 134.60, 133.05 (C=C), 130.24, 128.75 (2Ar), 128.37 (C=C), 121.95, 120.10, 118.04, 116.00 (2Ar), 113.28, 107.88, 105.75, 102.57, 55.84 (OMe). HRMS (ESI), calcd for C_23_H_20_N_2_O_5_ [M+H]^+^: 405.1420, found: 405.1418.

N′-((*E*)-2,4-dihydroxy-6-((*E*)-4-hydroxystyryl)benzylidene)-4-methoxybenzohydrazide (**6b**): Yield 70%; Brown solid. Mp 199–201 °C. ^1^H NMR (300 MHz, DMSO-d6) δ 12.46 (s, 1H), 11.83 (s, 1H), 8.97 (s, -CH=N-), 7.92 (d, *J* = 8.8 Hz, 2H), 7.48 (d, *J* = 8.5 Hz, 2H), 7.31 (d, *J* = 16.0 Hz, 1H), 7.08 (d, *J* = 8.8 Hz, 2H), 6.93 (d, *J* = 16.0 Hz, 1H), 6.80 (d, *J* = 8.5 Hz, 2H), 6.56 (d, *J* = 2.1 Hz, 1H), 6.26 (d, *J* = 2.1 Hz, 1H), 3.81 (s, 3H). ^13^C NMR (75 MHz, DMSO-d6) δ 162.59 (C=O), 162.15 (Ar-O), 161.15 (Ar-O), 160.74 (Ar-O), 158.16 (Ar-O), 147.93 (C=N), 140.83, 132.99 (C=C), 129.86 (2Ar), 128.73 (2Ar), 128.39 (C=C), 125.21, 121.98, 116.00 (2Ar), 114.29 (2Ar), 107.96, 105.68, 102.56, 55.91 (OMe). HRMS (ESI), calcd for C_23_H_20_N_2_O_5_ [M+H]^+^: 405.1418 found: 405.1415.

N′-((*E*)-2,4-dihydroxy-6-((*E*)-4-hydroxystyryl)benzylidene)-4-fluorobenzohydrazide (**6c**): Yield 55%; Yellow solid. Mp 175–177 °C. ^1^H-NMR (300 MHz, DMSO-d6) δ 10.08 (s, OH), 9.71 (s, OH), 8.97 (s, -CH=N-), 8.90 (s, OH), 8.00 (dd, *J* = 8.7, 5.5 Hz, 2H), 7.48 (d, *J* = 8.5 Hz, 2H), 7.39 (tapparent, *J* = 8.8 Hz, 2H), 7.31 (d, *J* = 16.1 Hz, 1H), 6.93 (d, *J* = 16.1 Hz, 1H), 6.80 (d, *J* = 8.5 Hz, 2H), 6.57 (d, *J* = 2.1 Hz, 1H), 6.27 (d, *J* = 2.1 Hz, 1H). ^13^C-NMR (75 MHz, DMSO-d6) δ 163.05 (C=O), 161.70 (Ar-F), 161.22 (Ar-O), 160.94 (Ar-O), 158.18 (Ar-O), 148.68 (C=N), 141.03, 133.10 (C=C), 130.75, 130.63, 128.75 (2Ar), 128.38 (C=C), 121.95, 116.22, 116.02 (2Ar), 115.93 (2Ar), 107.85, 105.81, 102.56. HRMS (ESI), calcd for C_22_H_17_FN_2_O_4_ [M+H]^+^: 392.2357 found: 392.2365.

N′-((*E*)-2,4-dihydroxy-6-((E)-4-hydroxystyryl)benzylidene)-2,3-dimethoxybenzohydrazide (**6d**): Yield 65%; Brown solid. Mp 215–217 °C. ^1^H NMR (300 MHz, DMSO-d6) δ 8.91 (s, -CH=N-), 7.45 (d, *J* = 8.6 Hz, 2H), 7.28 (d, *J* = 16.1 Hz, 1H), 7.24–7.20 (m, 2H), 7.17 (d, *J* = 8.0 Hz, 1H), 7.13 (d, *J* = 2.1 Hz, 1H), 6.92 (d, *J* = 16.0 Hz, 1H), 6.78 (d, *J* = 8.6 Hz, 2H), 6.58 (d, *J* = 2.1 Hz, 1H), 6.26 (d, *J* = 2.2 Hz, 1H), 3.85 (s, 3H), 3.79 (s, 3H). ^13^C NMR (75 MHz, DMSO-d6) δ 162.12 (C=O), 161.18 (Ar-O), 160.92 (Ar-O), 160.45 (Ar-O), 158.14 (Ar-O), 153.03 (Ar-O), 148.35 (C=N), 146.71, 140.90, 132.90 (C=C), 129.67 (2Ar), 128.70 (C=C), 128.36, 124.81, 121.72, 120.79, 115.97 (2Ar), 115.50, 107.73, 105.60, 102.53, 61.72 (OMe), 56.42 (OMe). HRMS (ESI), calcd for: C_24_H_22_N_2_O_6_ [M+H]^+^: 435.1550 found: 435.1552.

N′-((*E*)-2,4-dihydroxy-6-((*E*)-4-hydroxystyryl)benzylidene)-2,4-dimethoxybenzohydrazide (**6e**): Yield 70%; Yellow solid. Mp 243–245 °C. ^1^H-NMR (300 MHz, DMSO-d6) δ 12.53 (s, OH), 11.32 (s, OH), 8.98 (s, -CH=N-), 7.75 (d, *J* = 8.4 Hz, 1H), 7.48 (d, *J* = 8.2 Hz, 2H), 7.33 (d, *J* = 16.0 Hz, 1H), 6.95 (d, *J* = 16.0 Hz, 1H), 6.79 (d, *J* = 8.2, 2H), 6.72–6.63 (m, 3H), 6.59 (d, *J* = 2.4 Hz, 1H), 6.25 (d, *J* = 2.4 Hz, 1H), 3.93 (s, 3H), 3.84 (s, 3H). ^13^C-NMR (75 MHz, DMSO-d6) δ 163.64 (C=O), 161.47 (Ar-O), 161.24 (Ar-O), 160.71 (Ar-O), 159.02 (Ar-O), 158.13 (Ar-O), 147.92 (C=N), 140.70, 132.68 (C=C), 128.84 (2Ar), 128.45 (C=C), 121.83, 115.98 (2Ar), 114.65, 108.02, 106.27, 105.29, 102.59, 98.92, 56.58 (OMe), 56.04 (OMe). HRMS (ESI), calcd for: C_24_H_22_N_2_O_6_ [M+H]^+^: 435.1545 found: 435.1544.

N′-((*E*)-2,4-dihydroxy-6-((*E*)-4-hydroxystyryl)benzylidene)-2,5-dimethoxybenzohydrazide (**6f**): Yield 62%; Yellow solid. Mp 260–262 °C. ^1^H NMR (300 MHz, DMSO-d6) δ 12.40 (s, OH), 11.56 (s, OH), 8.98 (s, -CH=N-), 7.47 (d, *J* = 8.6 Hz, 3H), 7.31 (d, *J* = 16.0 Hz, 1H), 7.23 (d, *J* = 2.4 Hz, 1H), 7.12–7.08 (m, 2H), 6.94 (d, *J* = 16.0 Hz, 1H), 6.79 (d, *J* = 8.6 Hz, 2H), 6.59 (d, *J* = 2.2 Hz, 1H), 6.26 (d, *J* = 2.2 Hz, 1H), 3.85 (s, 3H), 3.75 (s, 3H). ^13^C NMR (75 MHz, DMSO-d6) δ 161.64 (C=O), 161.24 (Ar-O), 160.86 (Ar-O), 158.13 (Ar-O), 153.41 (Ar-O), 151.27 (Ar-O), 148.39 (C=N), 140.87, 132.82 (C=C), 128.78 (2Ar), 128.39 (C=C), 123.59, 121.77, 118.15, 115.97 (2Ar), 115.26, 113.83, 107.84, 105.47, 102.56, 56.84 (OMe), 56.03 (OMe). HRMS (ESI), calcd for: C_24_H_22_N_2_O_6_ [M+H]^+^: 435.1537 found: 435.1541.

N′-((*E*)-2,4-dihydroxy-6-((*E*)-4-hydroxystyryl)benzylidene)-3,4-dimethoxybenzohydrazide (**6g**): Yield 68%; Yellow solid. Mp 216–218 °C. ^1^H-NMR (300 MHz, DMSO-d6) δ 12.48 (s, OH), 11.82 (s, OH), 8.95 (s, -CH=N-), 7.57 (dd, *J* = 8.4, 2.0 Hz, 1H), 7.52–7.43 (m, 3H), 7.33 (d, *J* = 16.0 Hz, 1H), 7.10 (d, *J* = 8.5 Hz, 1H), 6.93 (d, *J* = 15.9 Hz, 1H), 6.80 (d, *J* = 8.4 Hz, 2H), 6.56 (d, *J* = 2.3 Hz, 1H), 6.26 (d, *J* = 2.3 Hz, 1H), 3.84 (s, 6H). ^13^C-NMR (75 MHz, DMSO-d6) δ 162.30 (C=O), 161.17 (Ar-O), 160.76 (Ar-O), 158.16 (Ar-O), 152.33 (Ar-O), 148.91 (Ar-O), 147.89 (C=N), 140.89, 133.00 (C=C), 128.77 (2Ar), 128.40 (C=C), 125.27, 122.09, 121.28, 116.01 (2Ar), 111.48, 111.24, 108.01, 105.71, 102.59, 56.49 (OMe), 56.12 (OMe). HRMS (ESI), calcd for: C_24_H_22_N_2_O_6_ [M+H]^+^: 435.1551 found: 435.1549.

N′-((*E*)-2,4-dihydroxy-6-((*E*)-4-hydroxystyryl)benzylidene)-3,5-dimethoxybenzohydrazide (**6h**): Yield 60%; Yellow solid. Mp 210–212 °C. ^1^H NMR (300 MHz, DMSO-d6) δ 12.40 (s, OHH), 11.89 (s, OHH), 8.96 (s, -CH=N-), 7.47 (d, *J* = 8.6 Hz, 2H), 7.32 (d, *J* = 16.0 Hz, 1H), 7.07 (d, *J* = 2.2 Hz, 2H), 6.93 (d, *J* = 16.0 Hz, 1H), 6.80 (d, *J* = 8.6 Hz, 2H), 6.73 (t, *J* = 2.2 Hz, 1H), 6.56 (d, *J* = 2.2 Hz, 1H), 6.26 (d, *J* = 2.2 Hz, 1H), 3.82 (s, 6H). ^13^C NMR (75 MHz, DMSO-d6) δ 162.33 (C=O), 161.23 (Ar-O), 160.94 (2Ar-O), 160.90 (Ar-O), 158.15 (Ar-O), 148.64 (C=N), 141.02, 135.22, 133.05 (C=C), 128.77 (2Ar), 128.37 (C=C), 121.98, 115.99 (2Ar), 107.88, 105.93, 105.75 (2Ar), 103.95, 102.55, 56.00 (OMe). HRMS (ESI), calcd for: C_24_H_22_N_2_O_6_ [M+H]^+^: 435.1550 found: 435.1552.

N′-((*E*)-2,4-dihydroxy-6-((*E*)-4-hydroxystyryl)benzylidene)isonicotinohydrazide (**7**): Yield 60%; Brown solid. Mp 232–234 °C. ^1^H NMR (300 MHz, DMSO-d6) δ 8.99 (s, -CH=N-), 8.80 (d, *J* = 6.0 Hz, 2H), 7.83 (d, *J* = 6.0 Hz, 2H), 7.48 (d, *J* = 8.6 Hz, 2H), 7.34 (d, *J* = 16.0 Hz, 1H), 6.93 (d, *J* = 16.0 Hz, 1H), 6.80 (d, *J* = 8.6 Hz, 2H), 6.57 (d, *J* = 2.2 Hz, 1H), 6.27 (d, *J* = 2.2 Hz, 1H). ^13^C NMR (75 MHz, DMSO-d6) δ 161.33 (C=O), 161.19 (Ar-O x 2), 158.20 (Ar-O), 150.89 (2Ar), 149.73 (C=N), 141.30, 140.34, 133.22 (C=C), 128.77 (2Ar), 128.35 (C=C), 121.96 (Ar), 121.85, 118.57, 116.01 (2Ar), 107.71, 106.94, 102.52. HRMS (ESI), calcd for C_21_H_17_N_3_O_4_ [M+H]^+^: 375.1253 found: 376.1251.

2-((*E*)-2,4-dihydroxy-6-((*E*)-4-hydroxystyryl)benzylidene)hydrazine-1-carbothioamide (**8**): Yield 50%; Yellow solid. Mp 242–244 °C. ^1^H NMR (600 MHz, DMSO-d6) δ 8.64 (s, -CH=N-), 8.02 (s, OH), 7.60 (s, OH), 7.40 (d, *J* = 8.5 Hz, 2H), 7.30 (d, *J* = 16.0 Hz, 1H), 6.84 (d, *J* = 16.0 Hz, 1H), 6.75 (d, *J* = 8.5 Hz, 2H), 6.54 (d, *J* = 2.0, 1H), 6.21 (d, *J* = 2.0 Hz, 1H), 5.71 (s, OH). ^13^C NMR (151 MHz, DMSO-d6) δ 176.61 (C=S), 159.95 (Ar-O), 159.03 (Ar-O), 157.45 (Ar-O), 144.27 (C=N), 140.35, 131.43 (C=C), 128.54, 128.03 (2Ar), 127.93 (C=C), 115.45 (2Ar), 107.84, 105.21, 101.71. HRMS (ESI), calcd for C_16_H_15_N_3_O_3_S [M+H]^+^: 330.09004, found: 330.0895.

### 2.2. Biological Activity Assays

#### 2.2.1. Cell Lines and Culture Medium

Human colon cancer cell lines (SW480 and SW620), together with nonmalignant cells (CHO-K1 and HaCaT) were used to study the biological activity. Cells were purchased from the European Collection of Authenticated Cell Cultures (ECACC, UK). Dulbecco’s Modified Eagle Medium (DMEM) containing heat-inactivated (56 °C) horse serum (10%), non-essential amino acids (1%) and antibiotics (penicillin/streptomycin, 1%) (Gibco Invitrogen, Carlsbad, CA, USA) was used to maintain the cells, reducing the horse serum to 3%, and, adding insulin (10 mg/mL), transferrin (5 mg/mL) and selenium (5 ng/mL) (ITS-defined medium; Gibco, Invitrogen, Carlsbad, CA, USA) for all the experiments [38,39].

#### 2.2.2. Cell Viability

The colorimetric test with Sulforhodamine B (SRB) was used to evaluate cell viability. Cell density was adjusted to seed 20.000 cells/well in 96-well tissue culture plates. Cells were first incubated for 24 h and then treated with the synthesized hybrids, the lead compounds (PIH and resveratrol), and the reference drug (5-FU) (concentrations between 0.01 and 200 µM), using DMSO (below 1%) as vehicle control. The optimum conditions were 37 °C and 5% CO_2_. After the end of each treatment, cold trichloroacetic acid (MERCK, 50% *v*/*v*) was added and preserved for one hour at 4 °C, to fix the cells. Then, cell proteins were stained with 0.4% (*w*/*v*) SRB (Sigma-Aldrich, St. Louis, MO, USA), and subsequently washed with acetic acid (1%) to remove unbound SRB. After air-drying, protein-bound SRB was solubilized in 10 mM Tris-base before reading the absorbance at 492 nm in a microplate reader (Mindray MR-96A) [40,41]. All experiments were performed at least three times.

#### 2.2.3. Antiproliferative Activity

The procedure for evaluating the antiproliferative activity was carried out using the same colorimetric test (SRB) described for cell viability, with minor modifications. Briefly, 2500 cells/well were seeded in 96-well tissue culture plates and incubated as in the previously described conditions for 0, 2, 4, 6, and 8 days. Cell lines were treated with increasing concentrations of the hybrids (between 3 and 48 µM; ranges were adjusted according to the IC_50_ values). The culture medium was changed every 48 h. After each incubation time, cells were fixed, stained, and read as previously described for this technique [42].

#### 2.2.4. Measurement of Mitochondrial Membrane Potential (ΔΨm)

To evaluate changes in mitochondrial membrane permeability, the fluorescent dyes DiOC6 (3,3′-dihexyloxacarbocyanine iodide, Thermo Fisher Scientific, Waltham, MA, USA) and propidium iodide (PI) were used. Cell density was adjusted to seed 2.5 × 10^5^ cells/well in 6-well tissue culture plates. Cells adhered for 24 h (37 °C and 5% CO_2_) and then, they were treated with hybrids **6e** and **7**, with their respective IC_50_ value, using DMSO (1%) as vehicle control. Subsequently, cells were scraped with the same culture mean and stained with both fluorescent dyes, incubating at room temperature for 30 min in darkness. For each sample, 10,000 events were analyzed through flow cytometry with excitation at 488 nm and detection of the emission with the green (530/15 nm) and the red (610/20 nm) filters. The results informed about the cells with depolarized mitochondrial membranes [41].

#### 2.2.5. Cell Cycle Analysis

Cell staining with the fluorescent propidium iodide (PI) was used to measure cell cycle distribution. The assays were carried out following the previous methodology described by Nicoletti et al. (1991). Briefly, 2.5 × 10^5^ cells/well were seeded in 6-well tissue culture plates at 37 °C and 5% CO_2_. After 24 h of adherence, cells were treated during 48 h with hybrids **6e** and **7**, together with the vehicle control. At the end of each treatment, cells were scraped, and the centrifuged pellet was resuspended in 1.8 mL of 70% ethanol at 4 °C to fix the cells overnight. Then, after centrifugation, cells were washed twice in versene buffer to completely remove the alcohol, besides, they were further resuspended in 300 µL of PBS containing 0.25 mg/mL RNAse (Type I-A, Sigma-Aldrich, Darmstadt, Germany) and 0.1 mg/mL PI. After the incubation in the dark (for 30 min at room temperature), 10,000 events were analyzed with a FACS Canto II flow cytometer and the software BD FACS Diva 6.1.3. (BD Biosciences, San Jose, CA, USA). The PI fluorescence signal was analyzed with excitation at 488 nm (using a Sapphire laser), and detection at 610 nm. The cell cycle model was fixed with FlowJo 7.6.2 (Ashland, OR, USA), applying the Dean-Jett-Fox model [43].

#### 2.2.6. Cell Death Induction by Resveratrol/Hydrazone Hybrids

Flow cytometric analysis using a double fluorescent staining with Annexin-V/FITC and propidium iodide (Roche Diagnostics) was used to evaluate phosphatidylserine exposure and membrane damage. Colon cancer cells (SW480 and SW620) were seeded at 2.5 × 10^5^ in each 6-well plate and treated for 48 h. The most active resveratrol/hydrazone hybrids were used and DMSO (1%) was included as a control. At the end of each treatment, cells were scraped and centrifuged. The pellet was resuspended in versene buffer with the fluorescent dyes and incubated for 20 min in darkness. Data were analyzed with FlowJo 7.6.2 (Ashland, OR, USA). Cells with double-positive staining for Annexin-V and PI were considered late apoptotic or dead cells, while the single staining with Annexin-V/FITC (Annexin-V/FITC positive) or propidium iodide (PI positive) was considered for early apoptotic or necrotic cells, respectively. Assays were performed twice [43].

#### 2.2.7. Statistical Analysis

GraphPad Prism software (version 7.04 for Windows, San Diego, CA, USA) was used for all statistical analyses. The experiments were performed at least twice, and data are reported as mean ± SE (standard error). The verification of normality was performed by the Shapiro-Wilk test. Analysis of variance between the control group (non-treated) and the treated one, was performed by one-way ANOVA followed by Dunnett’s test. Values with *p* ≤ 0.05 were considered significant.

## 3. Results and Discussion

### 3.1. Chemistry

**Scheme 1:**
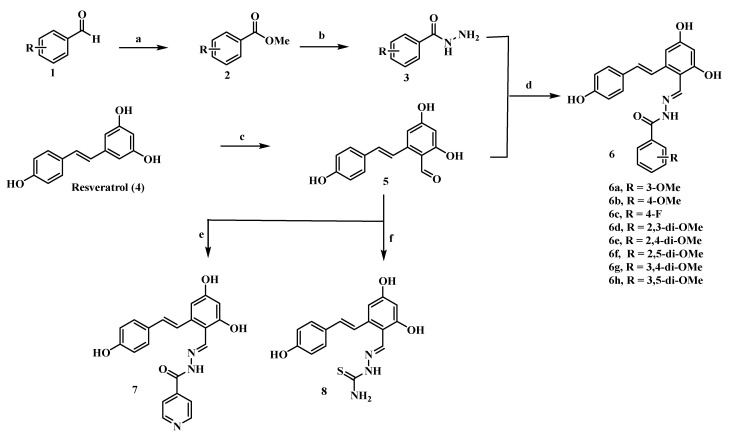
Synthesis of resveratrol-hydrazone hybrids. Reagents and conditions: (**a**) I_2_, KOH, MeOH, 0 °C, 71–84%; (**b**) N_2_H_4_, MeOH, MW, 60–90%; (**c**) POCl_3_, DMF, CH_3_CN, 90%; (**d**) EtOH-H_2_O, AcOH, US, 55–70%; (**e**) Isoniazid, EtOH-H_2_O, AcOH, US, 60%; (**f**) Thiosemicarbazide, EtOH-H_2_O, AcOH, US, 50%.

Synthesis of the hybrids began with the preparation of acylhydrazides from different benzoic acid esters. These esters **2** were obtained through the oxidation of aldehydes 1 [44] which were subsequently treated with hydrazine affording the compounds **3** [44,45], these compounds have been previously reported [46,47,48,49,50], however, our synthetic strategy involved mild reaction conditions in the esterification reactions and microwave-assisted reactions, in the obtention of acylhydracines, which allows to achieve the compounds with shorter reaction times than the conventional heating methods. Then acylhydrazines were coupled with resveratrol-aldehyde **5** yielding the hydrazones **6** in 55–70% [45,51]. Compound **5** was obtained by oxidation of resveratrol **4** with POCl_5_ [52]. Compounds **7** and **8** were synthesized by the reaction between aldehyde **5** with isoniazid and thiosemicarbazide, respectively (Scheme 1).

The structures of all compounds have been established by a combined study of ESI-MS, ^1^H-NMR, ^13^C-NMR, and ESI-MS spectra showed characteristic [M+H]^+^ peaks corresponding to their molecular weights. The assignments of all the signals to individual H or C-atoms have been performed on the basis of typical δ-values and J-constants. The ^1^H-NMR spectra of hybrids **6a**–**h** dissolved in DMSO showed signals of -CH=N- (~8.98 ppm) which appear as s, CH=CH (~7.30 and 6.90 ppm, as d every sign), OCH_3_ (~3.8 ppm) and Ar-H (~6.25–8.00 Ar-H). ^13^C-NMR spectra showed characteristic signals at ~148 ppm due to the presence of C=N, signals at ~163 ppm corresponding to the C=O group, and signals at ~133 and ~128 due to the C=C alkene group.

### 3.2. Biological Activity

#### 3.2.1. Effect of Hybrids Based on Resveratrol/Hydrazone on SW480, SW620, HaCaT, and CHO-K1 Cell Viability

The effect of the synthesized hybrids was evaluated in colorectal cancer cells (SW480 and SW620) and nonmalignant cell lines (HaCaT and CHO-K1). The parental compounds (PIH and resveratrol) and 5-fluorouracil (5-FU, the reference drug) were used as controls. Cytotoxicity was reported as 50% inhibitory concentration (IC_50_ values). Regarding the cytotoxic effect summarized in Table 1, all hybrids preserved the cytotoxic activity over time, as evidenced by the decrease in the IC_50_ value 48 h after treatment, being compounds **6e** and **7** (for SW480) and compound **7** (for SW620) the hybrids with better selectivity (SI >1) when tested in the nonmalignant cell lines. These selectivity indices were even better than those reported for the parental compounds and the reference drug, which were lower than 1 in both cell lines. As observed under the microscope, when cells were treated with the hybrid molecules, cellular morphology was perturbed by the treatment, showing changes in size and shape, while the vehicle control (DMSO) showed a typical and healthy shape. The effect caused for these hybrids was time- and concentration-dependent. Besides, there was a visible reduction in the number of cells suggesting either a cytotoxic or cytostatic effect on cancer cells. Other authors have reported similar findings regarding the parental compounds alone or in combination with other pharmacophores. Chalal et al. (2014) [53] synthesized a series of resveratrol derivatives (ferrocenylstilbene analogs) and evaluated their activity on cell viability of human hepatoblastoma HepG2 and human colorectal cancer SW480 cell lines, moreover, they also evaluated the biological activity using intestinal epithelial IEC-18 cells. These authors reported that the derivatives inhibited cancer cells and had a weaker effect on the HepG2 cell line, reporting high selectivity because of the lack of effect on nonmalignant cells (IEC-18). Similarly, Feng et al. (2016) [54] evaluated resveratrol alone in colon cancer cells and they reported that this stilbene significantly reduced cell viability of SW480, HT29, and HCA-17 72 h after treatment with 30 µM. Patil and colleagues (2018) [20] synthesized a series of small molecules based on hydrazide/hydrazone and reported that these molecules induced toxicity in a panel of colon cancer cells (SW620, HCT-116, and DLD-1) without showing toxicity in healthy L929 fibroblast cells. In addition, Narayanan et al. (2019) [55] recently reported the biological activity of thirteen thiazolyl hydrazone derivatives, using colon cancer cells (KM 12, HT-29, and COLO 205), showing one compound with better activity when compared with the conventional chemotherapeutic agent, Irinotecan, with IC_50_ values ranging between 0.41 ± 0.19 and 6.85 ± 1.44 µM. SAR analysis showed that the presence of an electron-withdrawing group in the 4-position of the aromatic ring (instead of an electron-donating group), improved the activity (**6c** vs. **6b**). This change was more noticeable if the electron donor group was in the 3-position (**6c** vs. **6a**). In addition, the double substitution in 2- and 4-positions in the aromatic ring showed these were essential to improve activity (**6e** vs. **6e** and **6f**). This effect was more evident in SW620 cells. Similarly, the double substitution in 3- and 5-positions of the aromatic ring were key to improving the activity (**6g** vs. **6h**). Finally, it was noticed that the bioisosteric modification led to an improvement in the activity in human colon cancer SW480 cell line. All these findings highlight the potential of these hybrid compounds to realize further experiments to explore new chemopreventive alternatives against colorectal cancer

#### 3.2.2. Antiproliferative Effect of Resveratrol/Hydrazone Hybrids on SW480 Cells

**Figure 3:**
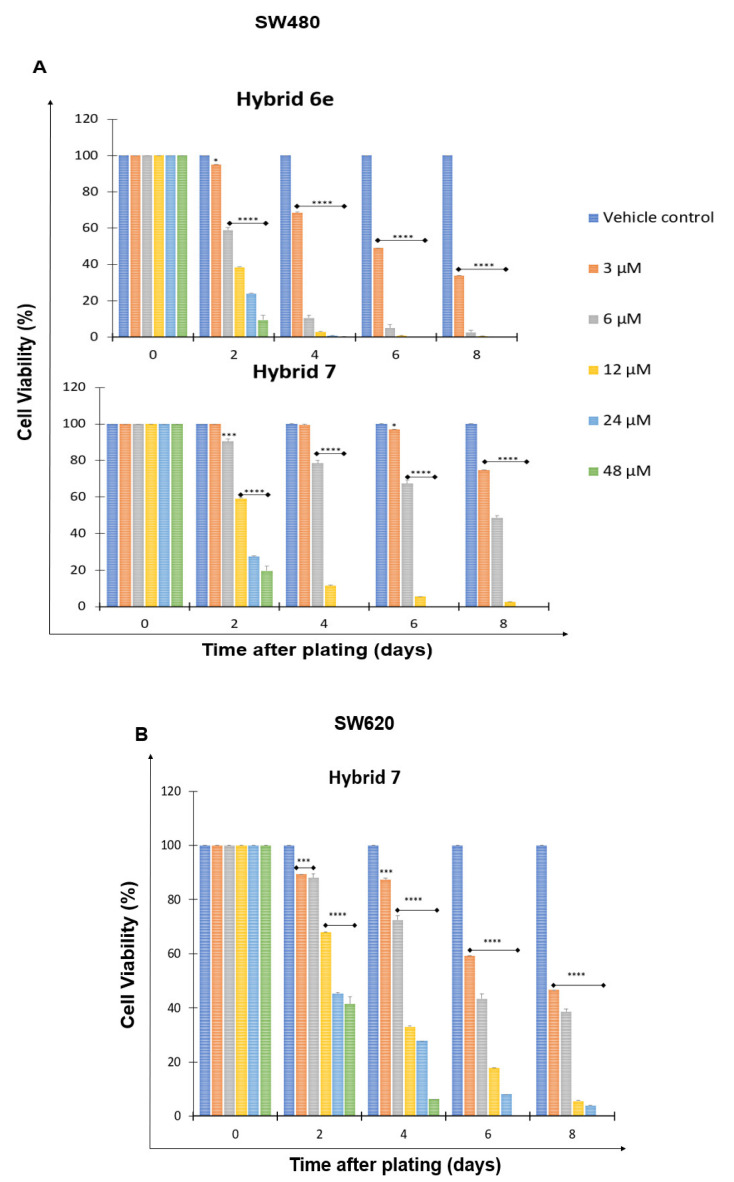
Antiproliferative effect of resveratrol/hydrazone hybrids on SW480 (**A**) and SW620 (**B**) cells. Results obtained with sulforhodamine B assay. DMSO 1% was used as a control. Data are presented as the mean ± SE of at least three independent experiments. * *p* < 0.05; *** *p* < 0.001; **** *p* < 0.0001.

**Figure 4:**
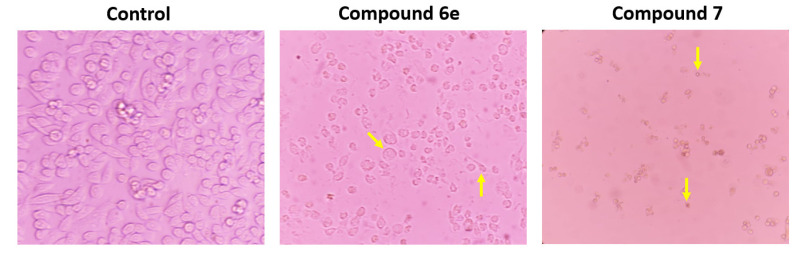
Representative images of SW480 cells 48 h after treatment with hybrid **6e** and **7**. Arrows indicate changes in size and shape. Magnification: 40×.

The most active hybrids (**6e** and **7**) were analyzed for longer periods to evaluate if they can display antiproliferative activity. After comparing each treatment with the control, the results indicate that the activity was time- and concentration-dependent (Table 2). Among the results obtained, when hybrid molecule **7** was evaluated in SW480 cells (Figure 3A), antiproliferative activity was observed from day 2, even at the lowest concentrations evaluated (3 µM). Similar results were observed when compounds **6e** and **7** were evaluated in SW620 cells at the same conditions (Figure 3B). Additionally, when cells were observed with an optical microscope, the cellular morphology of both cell lines was perturbed, showing changes in size and shape after the treatments with the hybrids (Figure 4). In addition, there was a clear reduction in the number of cells in comparison with the control, suggesting either a cytostatic or a cytotoxic effect. In a similar way, Ruan et al. (2011) [52] synthesized and characterized a series of resveratrol/chalcone derivatives, besides, they evaluated the effect of the new compounds in different cancer cells (A549, B16-F10 and HepG2) reporting that these hybrid molecules displayed better antiproliferative activity than the parental compound, resveratrol. These findings highlight the importance of evaluating these molecules using different models to make the best possible use of their potential.

#### 3.2.3. Changes in Mitochondrial Membrane Potential (ΔΨm) Induced by Resveratrol/Hydrazone Hybrids

**Figure 5:**
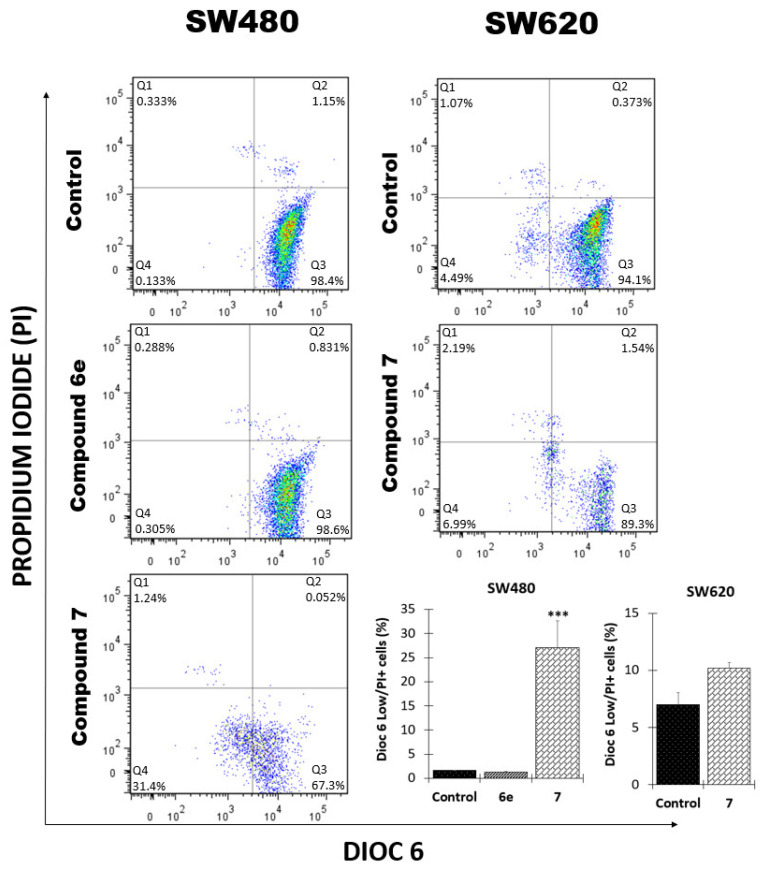
Mitochondrial membrane potential (ΔΨm) in SW480 and SW620 cells treated with either hybrids **6e**, **7**, or DMSO 1% (vehicle control). Q1 and Q2: PI-positive cells with membrane damage. Q3: DiOC6 high, live cells with high membrane polarization; Q4: DiOC6 low, cells in latency that lose membrane polarization. *** *p* < 0.001.

Mitochondrial dysfunction is determinant in the execution of cell death [56], thus, it is important to assess changes in Mitochondrial Membrane Potential (ΔΨm). We evaluated the effect of the most active resveratrol/hydrazone hybrids in SW480 and SW620 cells, using the carbocyanine fluorescent dye DiOC6. This dye accumulates in mitochondria due to its large negative membrane potential and it is released to the cytosol after a membrane depolarization (membrane with reduced ΔΨm), staining intracellular membranes [57,58]. According to the results (Figure 5), hybrid **7** was the only one with effect in SW480 cells, causing depolarization in the mitochondrial membrane regarding the control, which is evidenced by the increase in the DiOC6 low population. Other authors have also evaluated the mitochondrial effect of hybrid molecules using different compounds. Thus, Patil and colleagues (2018) [20] reported some hybrids based on hydrazide and hydrazone as inducers of mitochondrial outer membrane permeabilization, causing cell death mediated by the inhibition of antiapoptotic proteins such as Bcl-2, with the subsequent release of cytochrome c. These investigations reveal that the evaluated hybrid **7** could have chemopreventive potential playing its role in the mitochondrial disruption of colorectal cancer cells.

#### 3.2.4. Resveratrol/Hydrazone Hybrids Induce Cell Cycle Arrest on SW480 Cells

**Figure 6:**
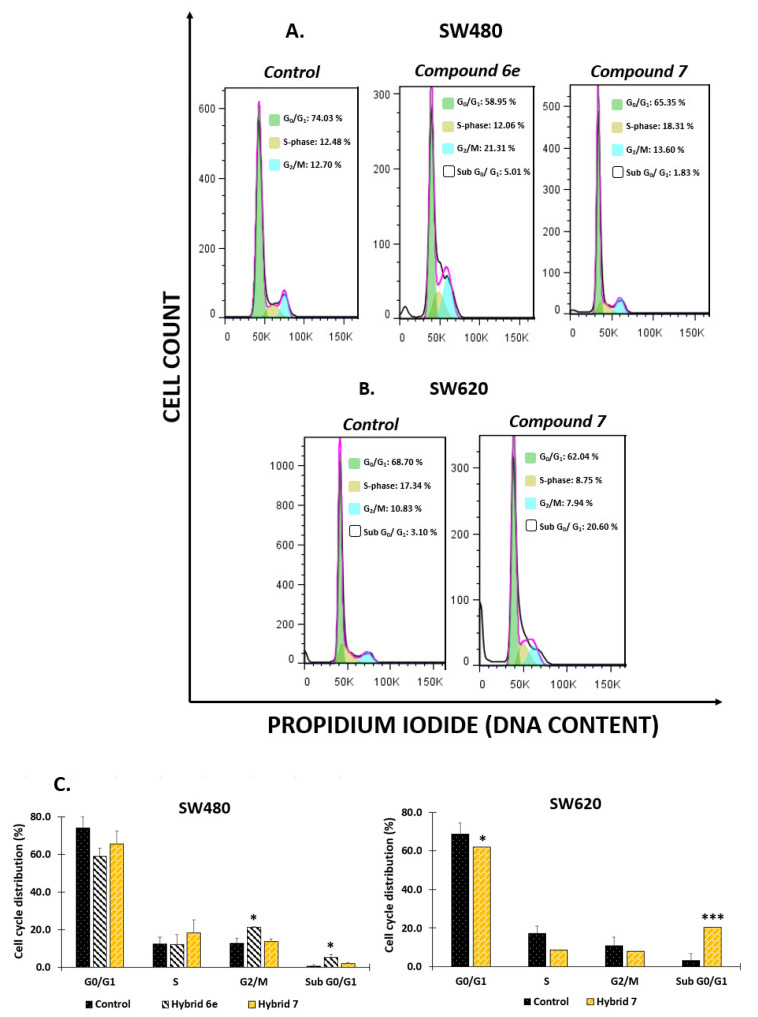
Effect of resveratrol/hydrazone hybrids on cell cycle distribution. (**A**) SW480 cells; (**B**) SW620 cells; (**C**) Statistical analysis of cell cycle phase distribution. One representative image of two independent experiments is shown. Data are presented as means ± SE of three independent tests. * *p* < 0.05 versus control, *** *p* < 0.001 versus control.

It has been suggested that different mutations in malignant cells contribute to changes in cell cycle distribution [59], thus, its modulation is seen as a possible target in the search for compounds with anticancer activity [60]. Because of this, the activities of hybrids **6e** and **7** were evaluated to determine the effect on cell cycle distribution of SW480 (Figure 6A) and SW620 (Figure 6B) colorectal cancer cells. These were treated for 48 h with the compounds. As shown in Figure 5, when the cells were evaluated through flow cytometry, it was observed that all hybrids induced changes in the normal distribution regarding the control. Hybrid **6e** caused arrest in SW480 cells at the G2/M phase (21.31%) with a reduction in the population of G0/G1 (58.95%) regarding the control. Besides, both compounds induced the formation of cells in the sub G0/G1 phase, which also was observed with hybrid **7** on SW620 cells (20.60%), suggesting cell death in this cell line at the conditions evaluated. In addition, according to the statistical analysis shown in Figure 6C, it was appreciated that the main changes induced by compound **6e** in SW480 cells and hybrid **7** in SW620 were statistically significant. Comparable results were reported by Patil et al. (2018) [20], who reported one hydrazide/hydrazone hybrid molecule with an effect on cell cycle distribution of colon cancer cells (HCT-116) 24 h post-treatment, causing arrest in the G2/M phase. Besides, Narayanan and colleagues (2019) [55] reported another Indanone-based thiazolyl hydrazone derivative that caused arrest at the G2/M phase of KM 12, HT-29, and COLO 205 cell lines in a concentration-dependent manner. Bai and colleagues (2009) [61] reported that resveratrol alone induced cell cycle arrest in the G1 stage of human bladder cancer cell line T24, mediated by the activation of p21 and downregulation of cyclin D1, phosphorylated Rb and cyclin-dependent kinase 4. Besides, Freitas Silva et al. (2018) [62] reported one resveratrol/curcumin derivative with the ability to arrest the cycle of MCF-7 cells at the G2/M stage, modulating nuclear kinase proteins which play a critical role in mitosis progression. These findings suggest that these resveratrol/hydrazone hybrids evaluated could be potential chemopreventive agents or possible adjuvants in conventional chemotherapy for different cancers, particularly colorectal cancer.

#### 3.2.5. Cell Death Induction by Resveratrol/Hydrazone Hybrids

**Figure 7:**
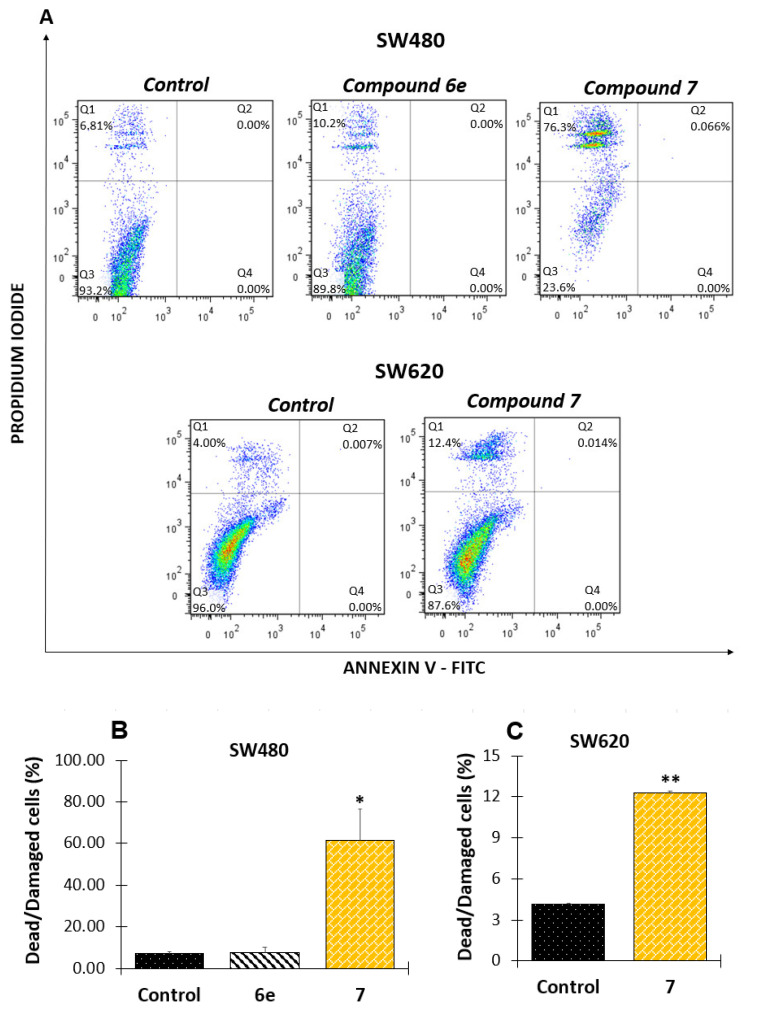
Flow cytometry analysis of apoptosis. (**A**) Representative histograms of SW480 and SW620 cells. Representation of data in bar chart form of (**B**) SW480 cells and (**C**) SW620 cells. Q1 + Q2: Cells which lost membrane integrity (Late apoptotic cells, dead cells, necroptotic cells, secondary necrotic cells, others); Q3: Viable cells; Q4: Early apoptotic cells. Control cells were treated with DMSO (1%). All experiments were performed in duplicate and gave similar results. *p* values lower than 0.05 were considered statistically significant (* *p* < 0.05; ** *p* < 0.01).

The plasma membrane acts as a direct barrier against the extracellular environment. It helps to maintain homeostasis and allows signal transduction and transport of molecules across the membrane. Considering these facts, the loss of membrane integrity puts an end to cellular life. Supported by the previous findings observed for mitochondrial membrane potential and cell cycle distribution, in this investigation we evaluated if the hybrids based on resveratrol/hydrazone induce plasma membrane disintegration and possibly cell death. Double staining with annexin-FITC and propidium iodide was used. The results are illustrated in Figure 6. After 48 h exposure, it was observed that hybrid **7** induced plasma membrane disintegration in both SW480 and SW620 cell lines (Figure 7), as evidenced by the displacement of the cells through the upper quadrant with positive staining for propidium. Similarly, in a previous study, it was reported that resveratrol alone induced apoptosis in colon cancer cells (HCA-17 and SW480) 48 h after treatment, modulating the expression of inflammatory cytokines [54]. In addition, Bai and colleagues (2009) [61] demonstrated that resveratrol cause apoptosis in human bladder cancer cells (T24) through the modulation of the antiapoptotic protein Bcl-2 and the activation of caspases-9 and -3, and the subsequent degradation of the poly (ADP-ribose) polymerase. Likewise, some hybrids based on resveratrol and aspirin were synthesized and evaluated by Salla and colleagues (2020) [63], who reported one compound with apoptotic activity mediated by inhibition of the nuclear factor kappa B (NF κB). On the other hand, the same pro-apoptotic effect induced by hybrids based on hydrazone has also been reported by different authors. Thus, Patil et al. (2018) [20] reported one hydrazide-hydrazone-based small molecule that caused caspase -9 and 3 cleavages in colon cancer cells (HCT-116). Moreover, it was reported an indanone-based thiazolyl hydrazone derivative that induced early and late apoptosis in a concentration-dependent manner in human colon cancer cell lines (HT-29, COLO 205, and KM 12) without inducing any significant necrosis in these cells [55]. Considering these findings and the multiple pathways involved in the apoptotic process, we hypothesize that the resveratrol/hydrazone hybrids evaluated could exert chemopreventive potential as evidenced by the in vitro cell death induction in SW480 and SW620 cell lines, however, it is necessary to carry out further studies.

## 4. Conclusions

We show for the first time that the synthesized hybrids **6e** and **7**, based on resveratrol/hydrazone, induce antiproliferative activity in two different adenocarcinoma cells (SW480 and SW620), probably involving selective cytotoxic activity and cell cycle arrest in the G2/M stage with the formation of cells in the subG0/G1 phase, suggesting cell death in these cell lines at the conditions evaluated. Besides, considering that hybrid **7** induced mitochondrial depolarization in SW480 cells and cell membrane damage in both cell lines evaluated, we hypothesize that this hybrid induces cell death in SW480 mediated by mitochondria. Our findings suggest that these hybrid compounds could be promising chemopreventive agents against colorectal cancer and thus, it would be interesting to carry out further studies.

## Figures and Tables

**Table 1:** Cytotoxic effect of resveratrol/hydrazone hybrids on SW480, SW620, HaCaT and CHO-K1 cell lines.

Hybrid Compound	24 H								48 H							
	IC_50_ ± SEM (µM)				Selectivity Index				IC_50_ ± SEM (µM)				Selectivity Index			
	CHO	HACAT	SW480	SW620	SI_1_	SI_2_	SI_3_	SI_4_	CHO	HACAT	SW480	SW620	SI_1_	SI_2_	SI_3_	SI_4_
**6a**	28.4 ± 1.48	47.5 ± 1.15	33.0 ± 2.13	172.1 ± 9.0	0.86	1.44	0.16	0.28	13.0 ± 1.3	2.2 ± 0.2	24.9 ± 2.8	41.5 ± 1.2	0.52	0.09	0.31	0.05
**6b**	23.4 ± 1.83	36.9 ± 3.53	36.1 ± 1.97	159.9 ± 9.4	0.65	1.02	0.15	0.23	14.4 ± 1.7	13.9 ± 0.7	11.3 ± 2.3	37.6 ± 2.9	1.28	1.24	0.38	0.37
**6c**	22.6 ± 2.38	32.8 ± 1.33	29.2 ± 3.55	57.7 ± 3.6	0.77	1.12	0.39	0.57	12 ± 1.6	14.6 ± 0.8	10.8 ± 2.0	12.5 ± 1.0	1.11	1.35	0.96	0.76
**6d**	35.5 ± 2.36	49.7 ± 1.96	34.8 ± 3.31	170.6 ± 8.7	1.02	1.43	0.21	0.29	22.4 ± 2.7	2.95 ± 0.2	20.5 ± 1.9	49.9 ± 2.1	1.09	0.14	0.45	0.06
**6e**	20.6 ± 3.06	34.4 ± 3.96	24.2 ± 3.29	74.8 ± 12.9	0.85	1.42	0.28	0.46	18.8 ± 1.9	19.1 ± 0.8	**6.5 ± 1.9**	27.8 ± 3.9	**2.89**	**2.94**	0.67	0.69
**6f**	22.7± 2.25	42.2 ± 5.9	23.4 ± 1.31	201.5 ± 2.5	0.97	1.80	0.11	0.21	15.9 ± 1.6	14.9 ± 1.3	12.2 ± 1.2	99.4 ± 6.9	1.30	1.22	0.16	0.15
**6g**	33.2 ± 1.99	63.7 ± 5.27	37.0 ± 2.24	190.6 ± 13.0	0.90	1.72	0.17	0.33	17.2 ± 2.0	17.7 ± 0.5	20.2 ± 2.7	40.3 ± 2.7	0.85	0.88	0.43	0.44
**6h**	22.8 ± 1.31	43.2 ± 4.02	29.1 ± 1.31	126.3 ± 20.9	0.78	1.49	0.18	0.34	14.0 ± 1.2	4.5 ± 0.6	7.2 ± 2.0	26 ± 3.2	1.95	0.62	0.54	0.17
**7**	67.2 ± 4.32	79.0 ± 6.73	84.2 ± 10.02	122.5 ± 10.8	0.80	0.94	0.55	0.64	40.5 ± 2.4	47.3 ± 2.5	**19.0 ± 1.4**	**38.41 ± 3.3**	**2.14**	**2.49**	**1.06**	**1.52**
**8**	41.9 ± 2.83	60.15 ± 3.99	41.0 ± 3.93	101.9 ± 3.5	1.02	1.47	0.41	0.59	36.7 ± 3.8	31.8 ± 1.4	41.2 ± 2.3	64.14 ± 2.9	0.89	0.77	0.57	0.50
**PIH**	308.5 ± 34.71	240.7 ± 16.55	281.4 ± 27.5	202.0 ± 2.0	1.10	0.86	1.53	1.19	75.3 ± 6.4	100.9 ± 10.8	111.6 ± 5.9	119.2 ± 7.0	0.67	0.90	0.63	0.85
**RESV**	118.4 ± 8.54	228.5 ± 18.25	153.6 ± 10.6	549.0 ± 38.5	0.77	1.49	0.22	0.42	64.0± 8.9	20.2 ± 2.7	123 ± 4.5	143.1 ± 4.0	0.52	0.16	0.45	0.14
**5-FU**	543.5 ± 52.94	>1000	1544 ± 127.9	898.8 ± 60.7	0.35	>1	0.59	>1	173.2 ± 14.6	118.7 ± 2.8	174.3 ± 19.1	180.9 ± 18.8	0.99	0.68	0.96	0.66

IC_50_ values were obtained from dose response curves for each compound. Selectivity index was determined as a ratio of the IC_50_ against nonmalignant cells (CHO K1 or HaCaT) to the IC_50_ for malignant cells (SW480 or SW620). S1: CHO-K1/SW480; S2: HaCaT/SW480; S3: CHO-K1/SW620; S4: HaCaT/SW620.

**Table 2:** Data of viability (%) different concentrations evaluated.

Cell Line	Compound	Concentration (µM)	Time after Plating (Days)			
			2	4	6	8
			Viability (%) ± SE			
**SW480**	**Hybrid 6e**	3	95.00 ± 3.11	68.46 ± 1.31	49.02 ± 2.08	33.86 ± 0.30
		6	58.90 ± 0.89	10.50 ± 0.87	5.03 ± 0.19	2.52 ± 0.10
		12	38.44 ± 2.67	2.78 ± 0.42	0.76 ± 0.36	0.48 ± 0.11
		24	23.85 ± 0.82	0.81 ± 0.38	0.00 ± 0.00	0.00 ± 0.00
		48	9.44 ± 1.17	0.30 ± 0.33	0.00 ± 0.00	0.00 ± 0.00
	**Hybrid 7**	3	100.0 ± 0.00	99.45 ± 0.55	96.87 ± 0.14	74.76 ± 0.02
		6	90.45 ± 1.38	78.51 ± 1.56	67.42 ± 1.82	48.49 ± 1.11
		12	59.10 ± 0.27	11.39 ± 0.35	5.49 ± 0.05	2.52 ± 0.22
		24	27.20 ± 0.50	0.00 ± 0.00	0.00 ± 0.00	0.00 ± 0.00
		48	19.65 ± 2.58	0.00 ± 0.00	0.00 ± 0.00	0.00 ± 0.00
**SW620**	**Hybrid 7**	3	89.35 ± 1.65	87.29 ± 0.75	59.25 ± 2.77	46.55 ± 0.03
		6	88.01 ± 2.48	72.42 ± 0.90	43.35 ± 0.48	38.53 ± 0.28
		12	67.82 ± 0.40	33.05 ± 1.80	17.82 ± 0.30	5.53 ± 0.11
		24	45.21 ± 0.38	27.84 ± 2.96	8.13 ± 1.48	3.93 ± 0.59
		48	41.47 ± 3.30	6.31 ± 0.74	0.26 ± 0.07	0.00 ± 0.00

## Data Availability

Data is contained within the article.

## Supplementary Materials

The following supporting information can be downloaded at: https://www.mdpi.com/article/10.3390/pharmaceutics14112278/s1, S1: ^1^H, ^13^C NMR and MS spectra, S2: HPLC analysis, of all hybrids.
