# Supplementary material for: Resveratrol/Hydrazone Hybrids: Synthesis and Chemopreventive Activity against Colorectal Cancer Cells
Source: Pharmaceutics. 2022 Oct 24;14(11):2278. doi: 10.3390/pharmaceutics14112278 (PMC9694494; doi:10.3390/pharmaceutics14112278)
Supplement: Supplementary file 1 [file pharmaceutics-14-02278-s001.zip › pharmaceutics-1967423-supplementary.pdf]

Supplementary Data

Resveratrol/hydrazone Hybrids: Synthesis and Chemopreventive Activity against Colorectal Cancer Cells

^{1}H, ^{13}C NMR and MS spectra	**S1**
HPLC analysis	**S2**

S1. ^{1}H, ^{13}C NMR and MS spectra

N'-((*E*)-2,4-dihydroxy-6-((E)-4-hydroxystyryl)benzylidene)-3-methoxybenzohydrazide (**6a**)

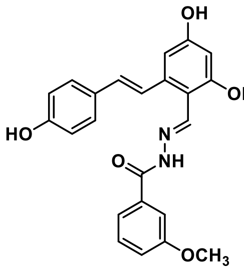

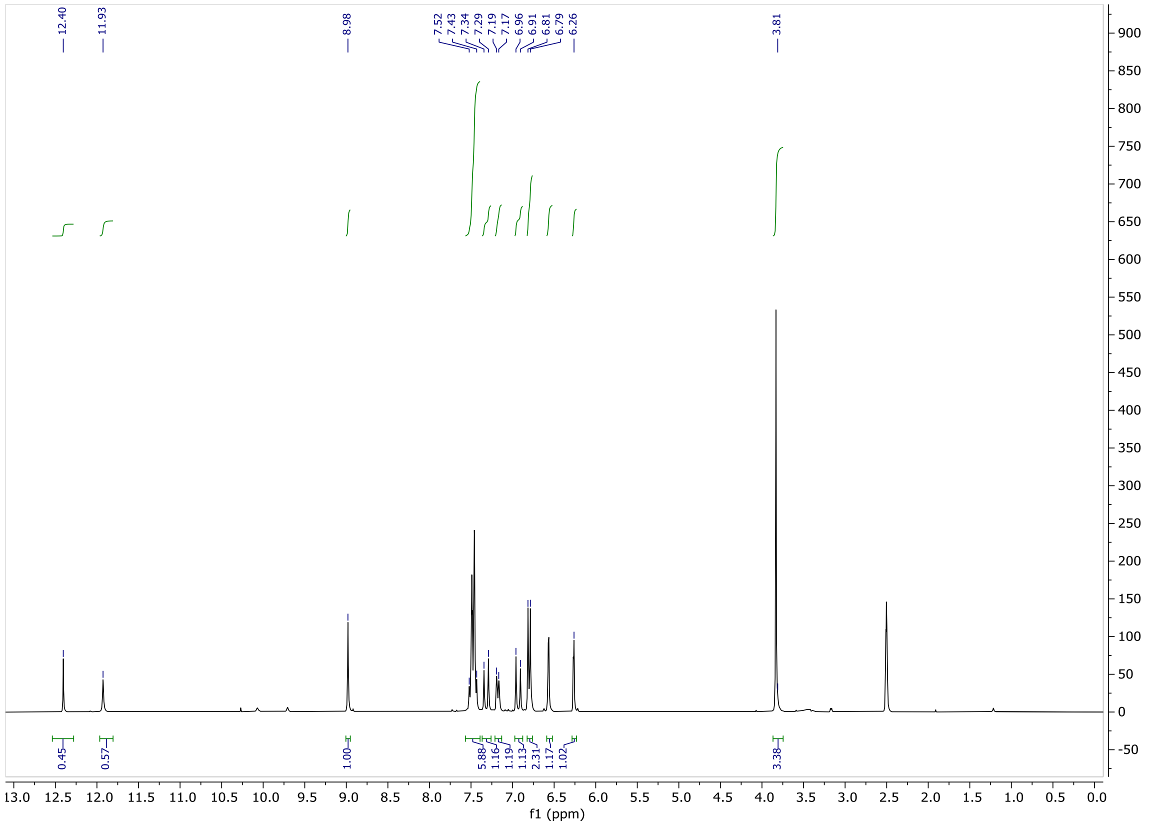

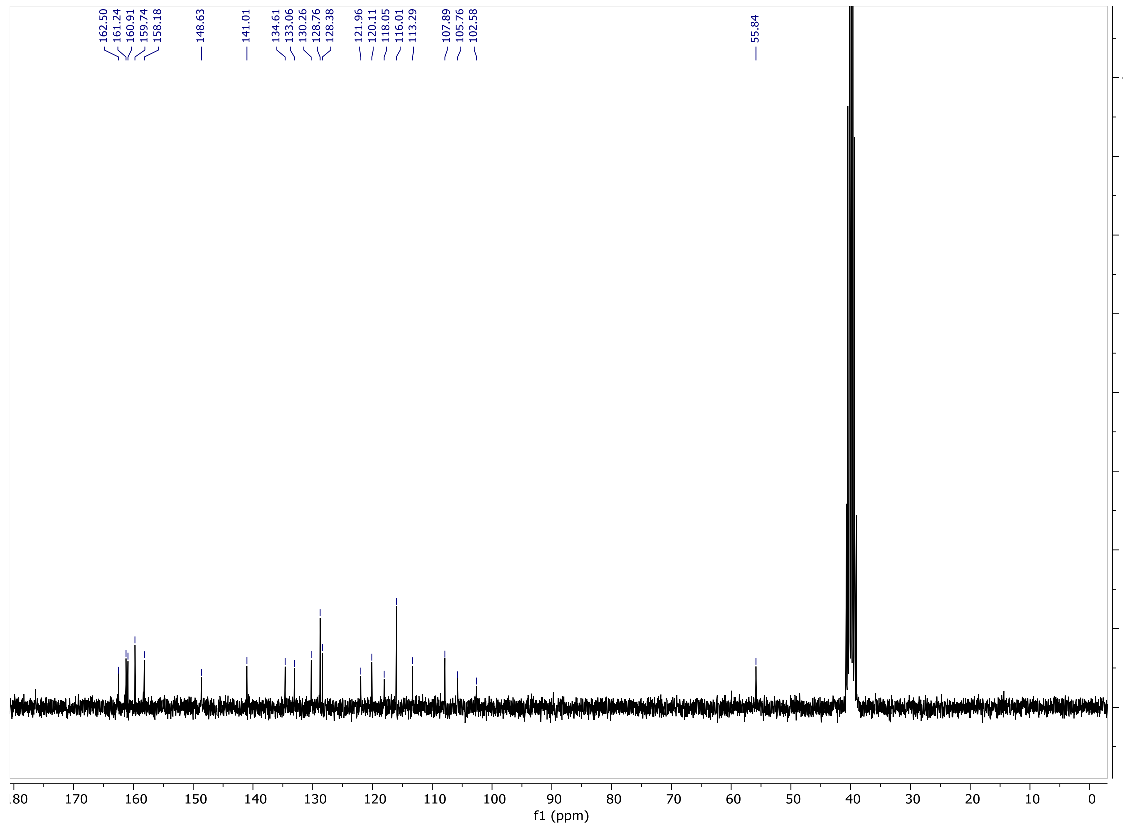

162.50
161.24
160.91
159.74
158.18
148.63
141.01
134.61
133.06
130.26
128.76
128.38
121.96
120.11
118.05
116.01
113.29
107.89
105.76
102.58
55.84
f1 (ppm)

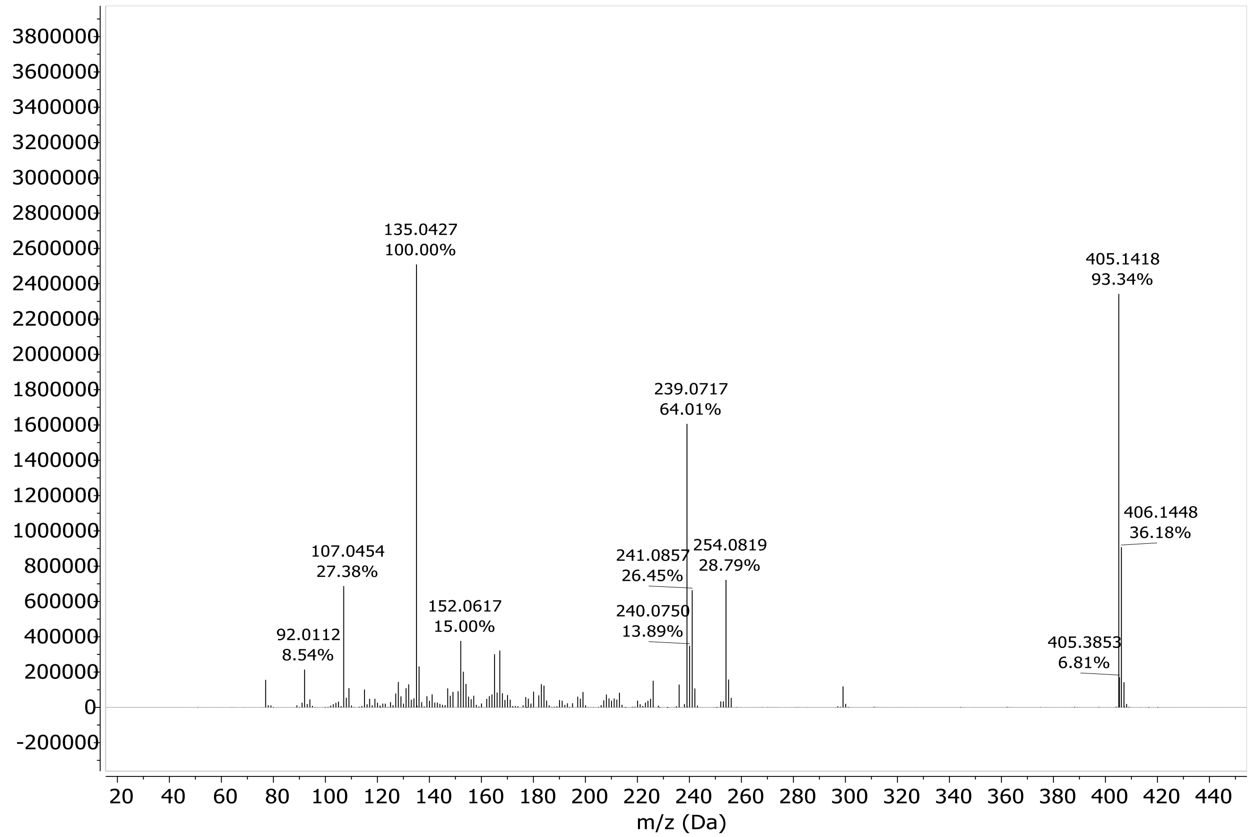

135.0427
100.00%
405.1418
93.34%
239.0717
64.01%
406.1448
36.18%
241.0857
26.45%
254.0819
28.79%
107.0454
27.38%
152.0617
15.00%
240.0750
13.89%
92.0112
8.54%
405.3853
6.81%
m/z (Da)

N′-((*E*)-2,4-dihydroxy-6-((*E*)-4-hydroxystyryl)benzylidene)-4-methoxybenzohydrazide (**6b**)

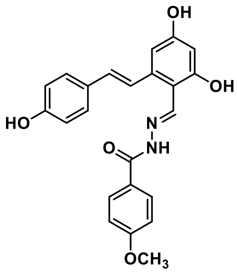

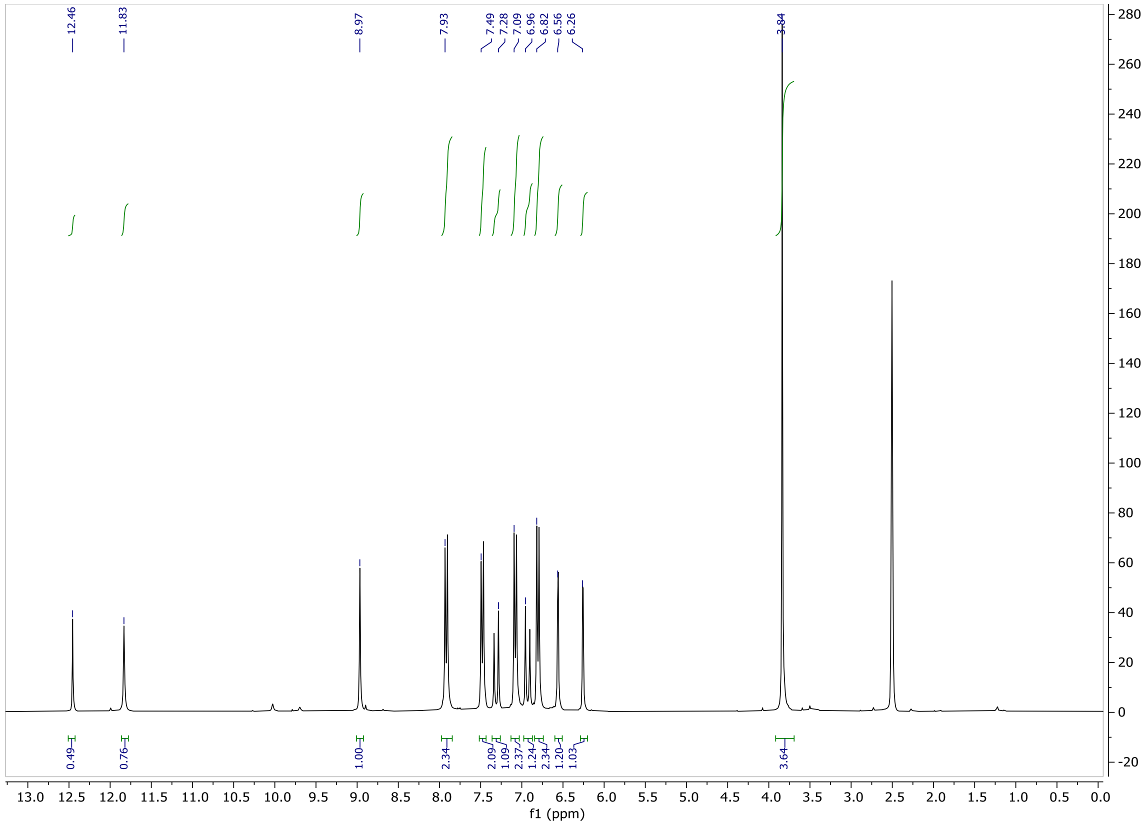

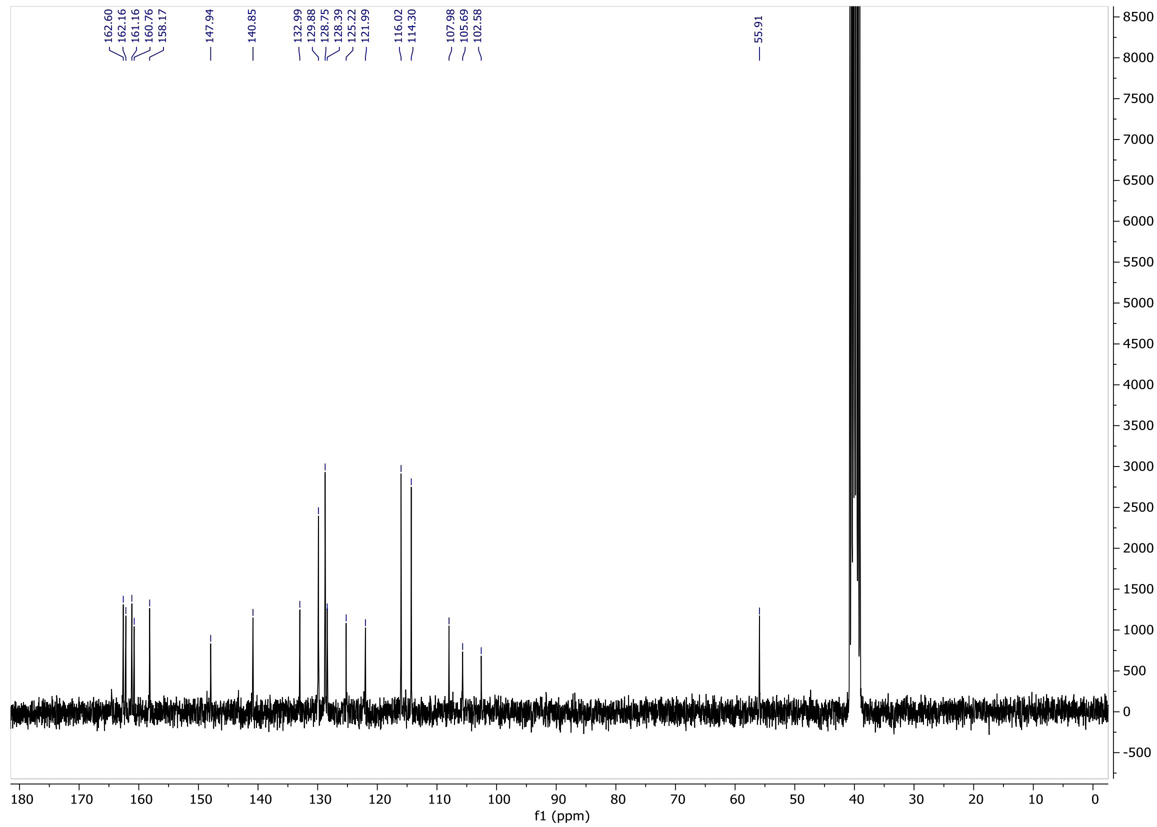
162.60
162.16
161.16
160.76
158.17
147.94
140.85
132.99
129.88
128.75
128.39
125.22
121.99
116.02
114.30
107.98
105.69
102.58
55.91
f1 (ppm)

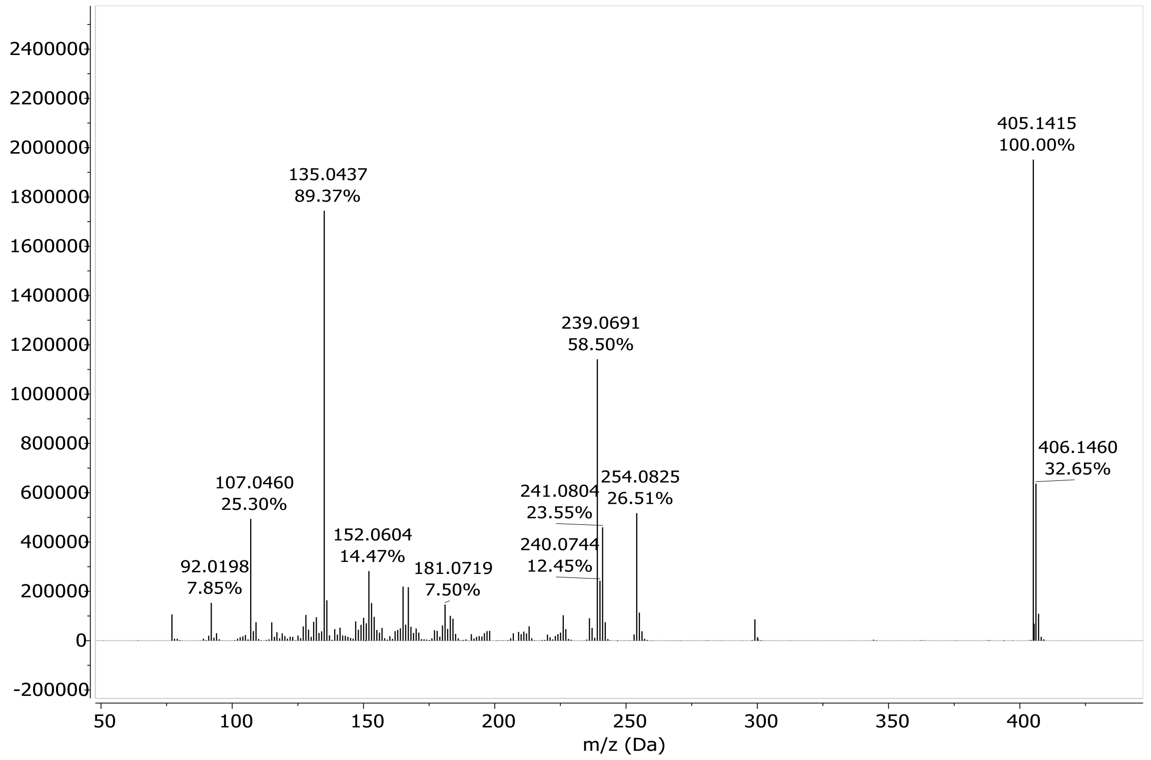
405.1415
100.00%
135.0437
89.37%
239.0691
58.50%
406.1460
32.65%
107.0460
25.30%
241.0804
23.55%
254.0825
26.51%
152.0604
14.47%
240.0744
12.45%
92.0198
7.85%
181.0719
7.50%
m/z (Da)

N'-((*E*)-2,4-dihydroxy-6-((*E*)-4-hydroxystyryl)benzylidene)-4-fluorobenzohydrazide (**6c**)

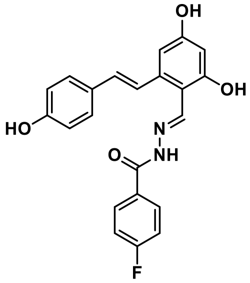

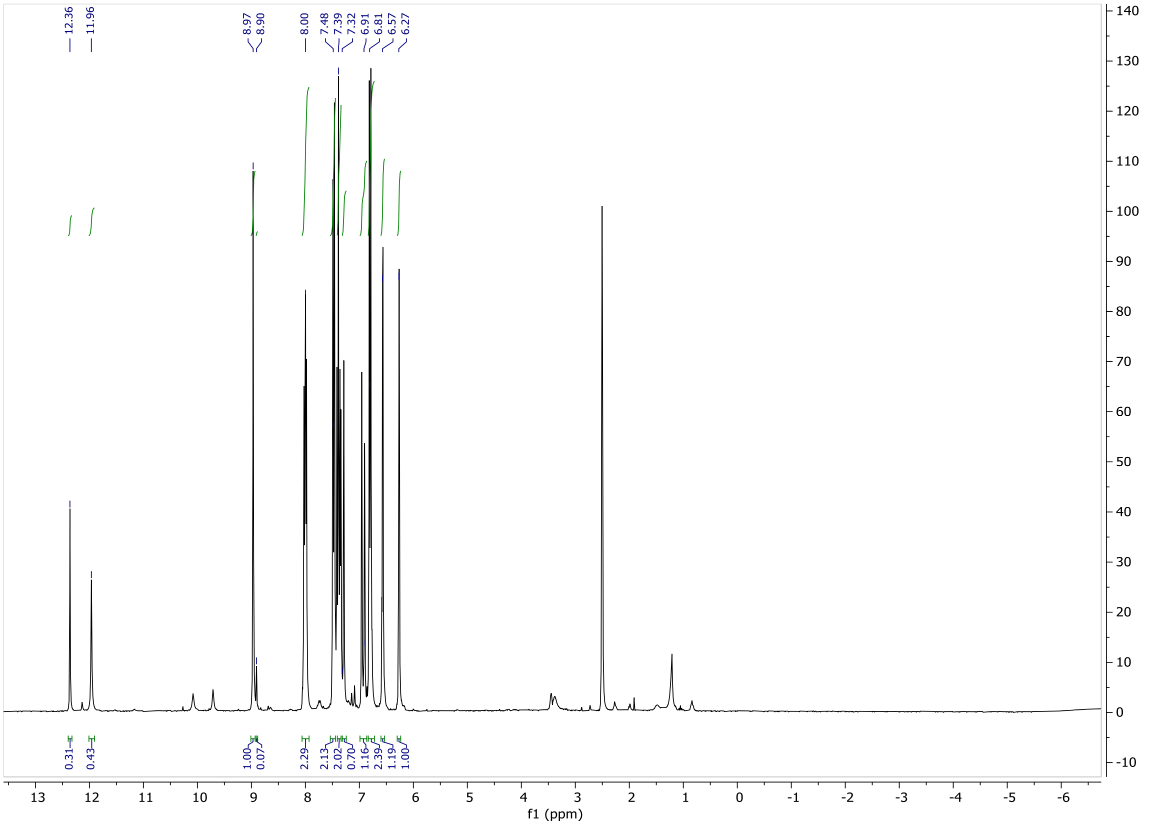

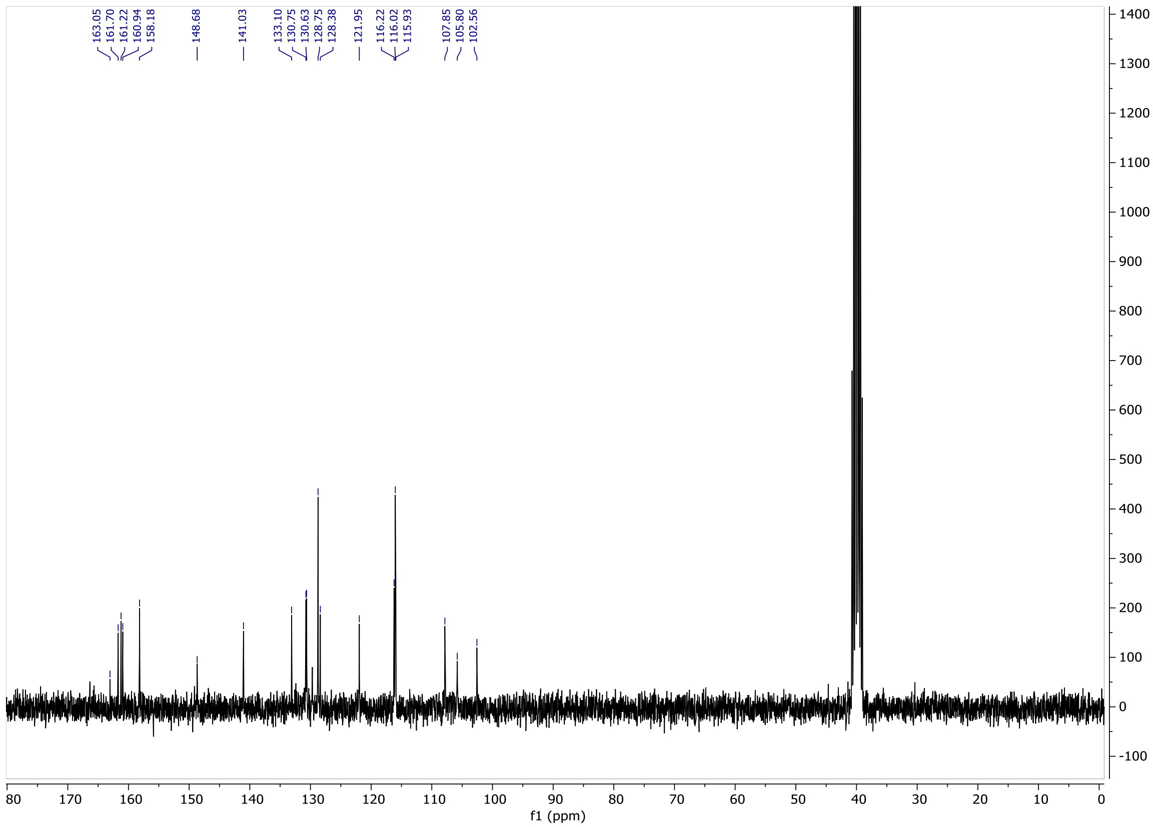
163.05
161.70
161.22
160.94
158.18
148.68
141.03
133.10
130.75
130.63
128.75
128.38
121.95
116.22
116.02
115.93
107.85
105.80
102.56
f1 (ppm)

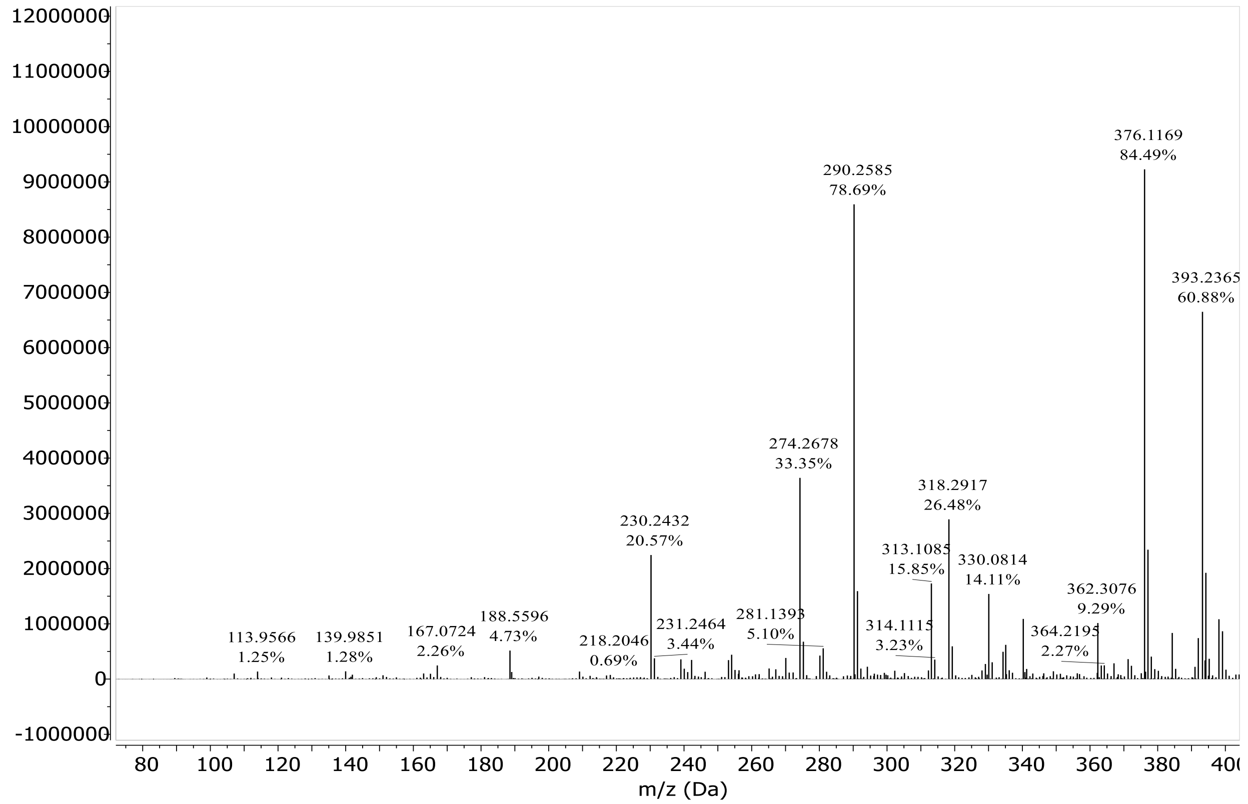
376.1169
84.49%
290.2585
78.69%
393.2365
60.88%
274.2678
33.35%
318.2917
26.48%
230.2432
20.57%
313.1085
15.85%
330.0814
14.11%
362.3076
9.29%
113.9566
1.25%
139.9851
1.28%
167.0724
2.26%
188.5596
4.73%
218.2046
0.69%
231.2464
3.44%
281.1393
5.10%
314.1115
3.23%
364.2195
2.27%
m/z (Da)

N'-((*E*)-2,4-dihydroxy-6-((*E*)-4-hydroxystyryl)benzylidene)-2,3-dimethoxybenzohydrazide (**6d**)

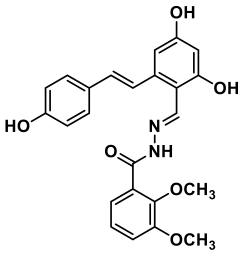

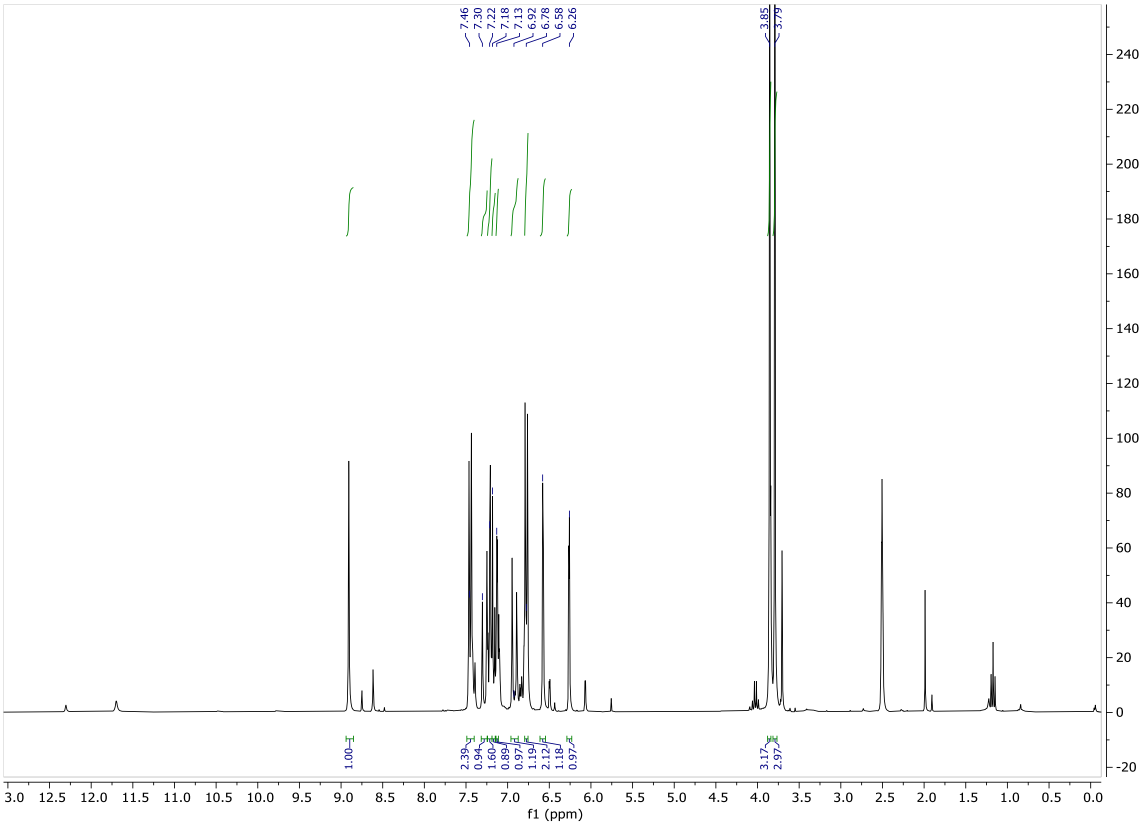

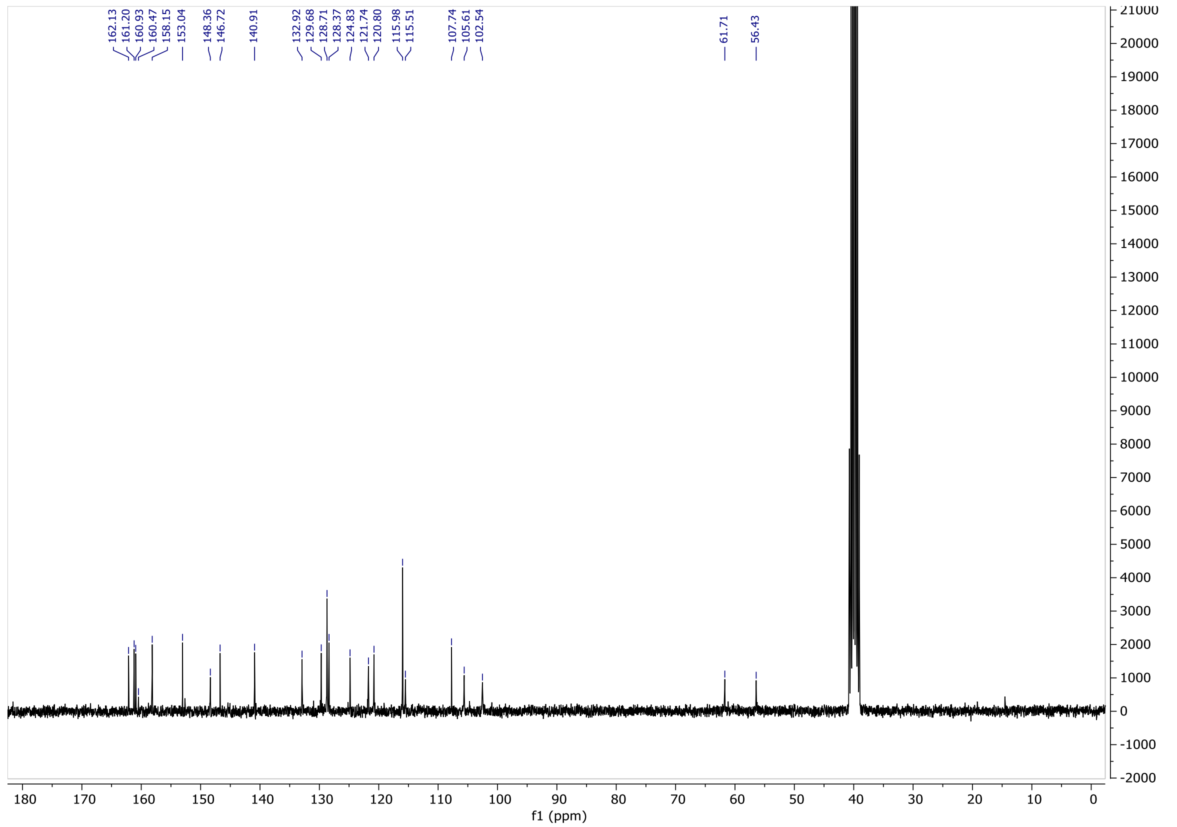
162.13
161.20
160.93
160.47
158.15
153.04
148.36
146.72
140.91
132.92
129.68
128.71
128.37
124.83
121.74
120.80
115.98
115.51
107.74
105.61
102.54
61.71
56.43
f1 (ppm)

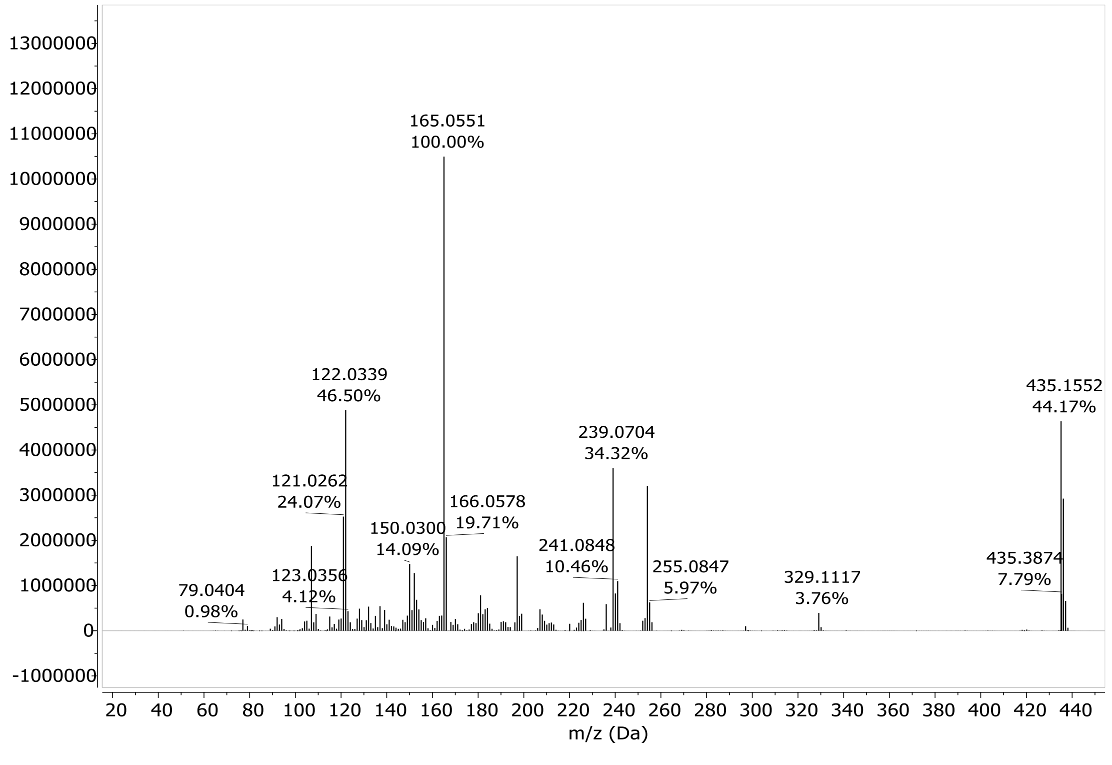
165.0551
100.00%
122.0339
46.50%
435.1552
44.17%
239.0704
34.32%
121.0262
24.07%
166.0578
19.71%
150.0300
14.09%
241.0848
10.46%
255.0847
5.97%
329.1117
3.76%
435.3874
7.79%
123.0356
4.12%
79.0404
0.98%
m/z (Da)

N'-((*E*)-2,4-dihydroxy-6-((*E*)-4-hydroxystyryl)benzylidene)-2,4-dimethoxybenzohydrazide (**6e**)

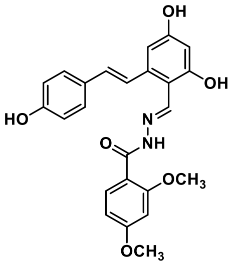

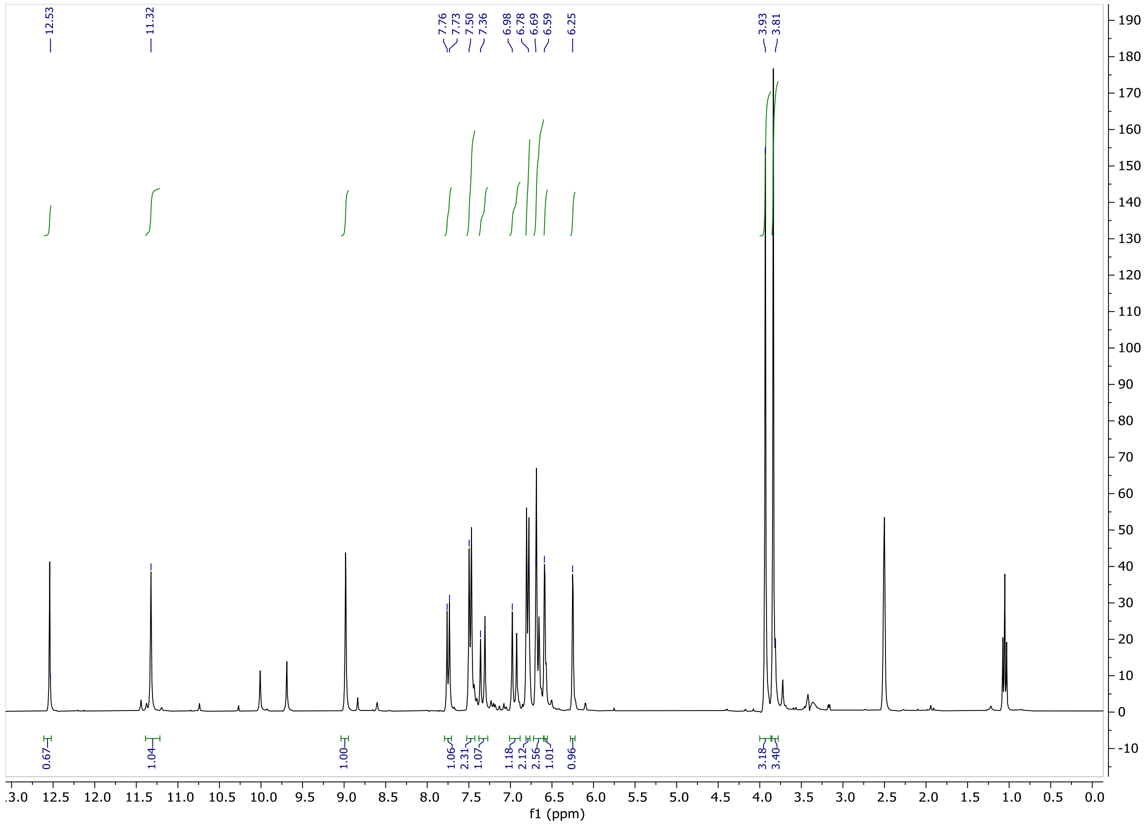

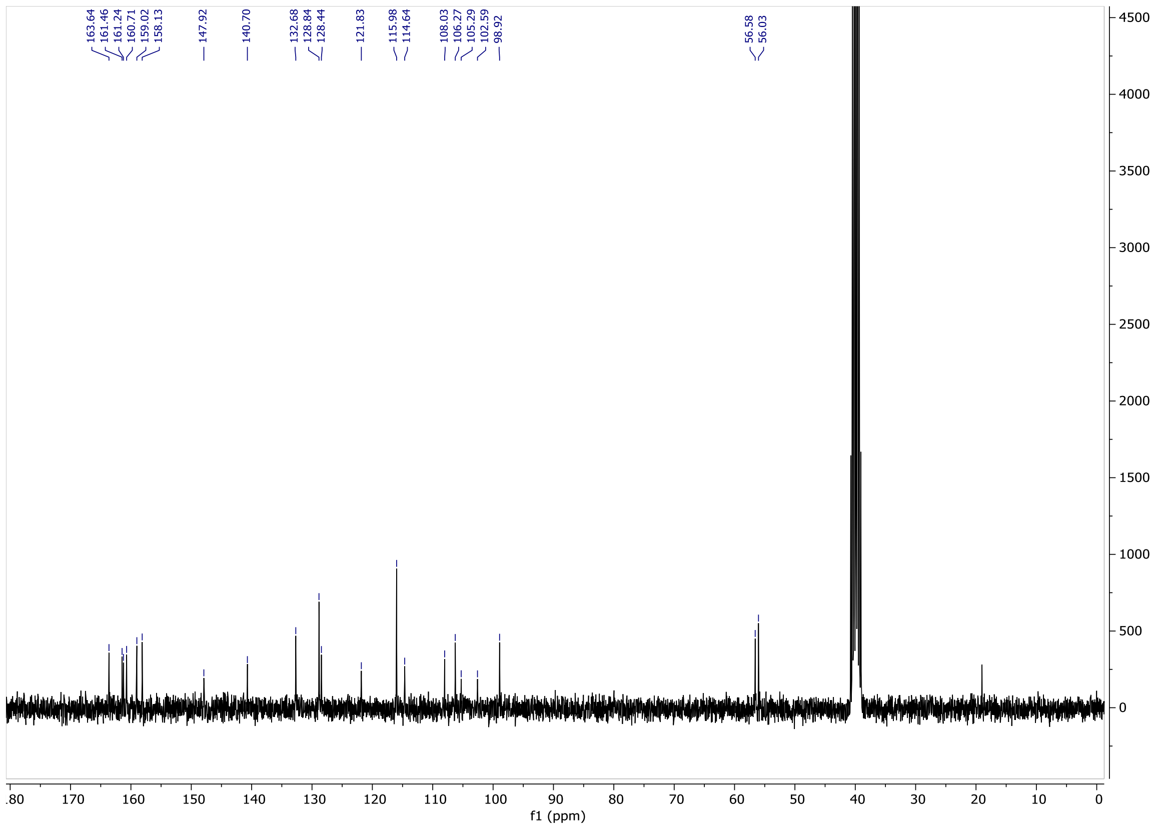
163.64
161.46
161.24
160.71
159.02
158.13
147.92
140.70
132.68
128.84
128.44
121.83
115.98
114.64
108.03
106.27
105.29
102.59
98.92
56.58
56.03
f1 (ppm)

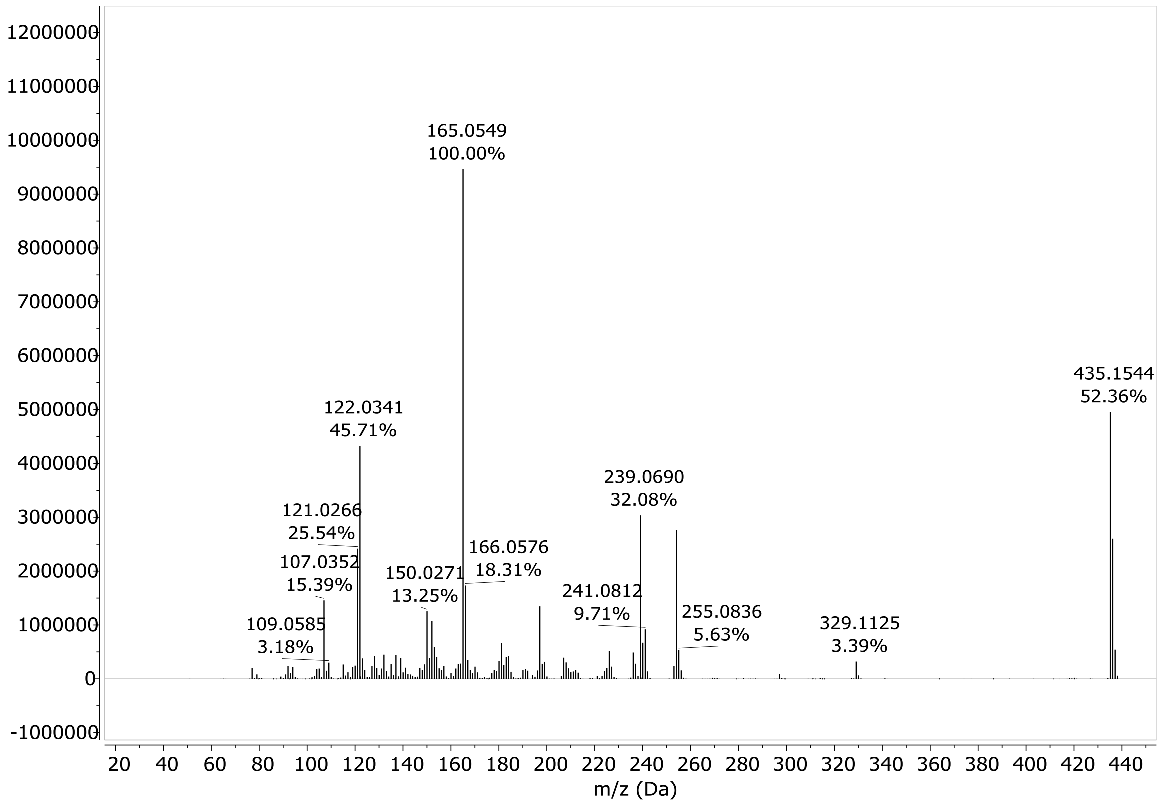
165.0549
100.00%
435.1544
52.36%
122.0341
45.71%
239.0690
32.08%
121.0266
25.54%
166.0576
18.31%
107.0352
15.39%
150.0271
13.25%
241.0812
9.71%
255.0836
5.63%
109.0585
3.18%
329.1125
3.39%
m/z (Da)

N'-((*E*)-2,4-dihydroxy-6-((*E*)-4-hydroxystyryl)benzylidene)-2,5-dimethoxybenzohydrazide (**6f**)

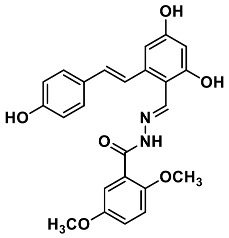

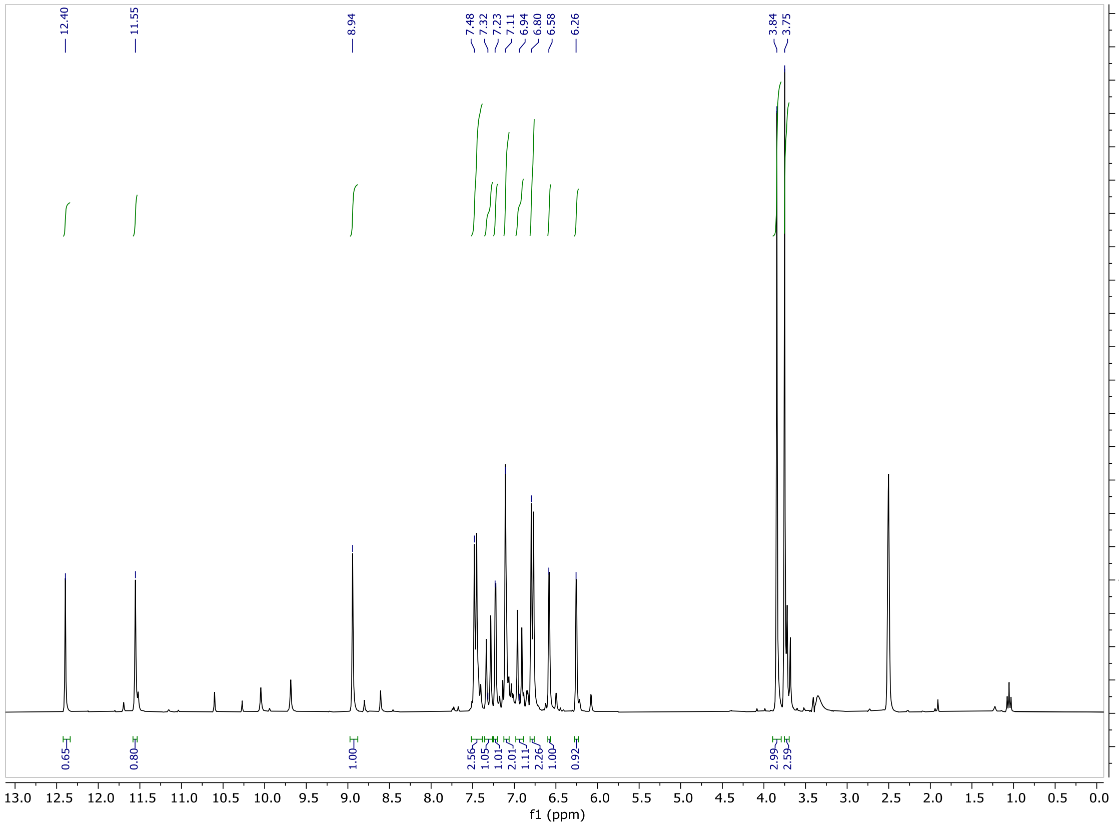

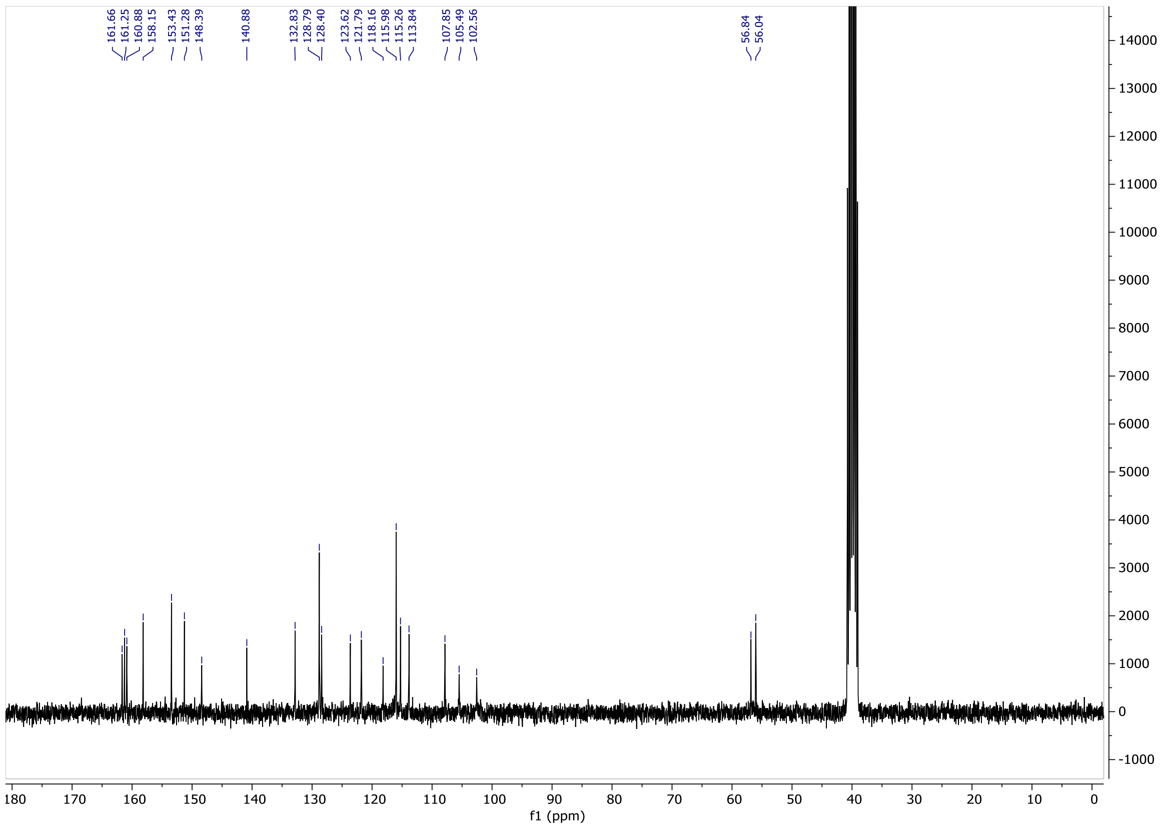
161.66
161.25
160.88
158.15
153.43
151.28
148.39
140.88
132.83
128.79
128.40
123.62
121.79
118.16
115.98
115.26
113.84
107.85
105.49
102.56
56.84
56.04
f1 (ppm)

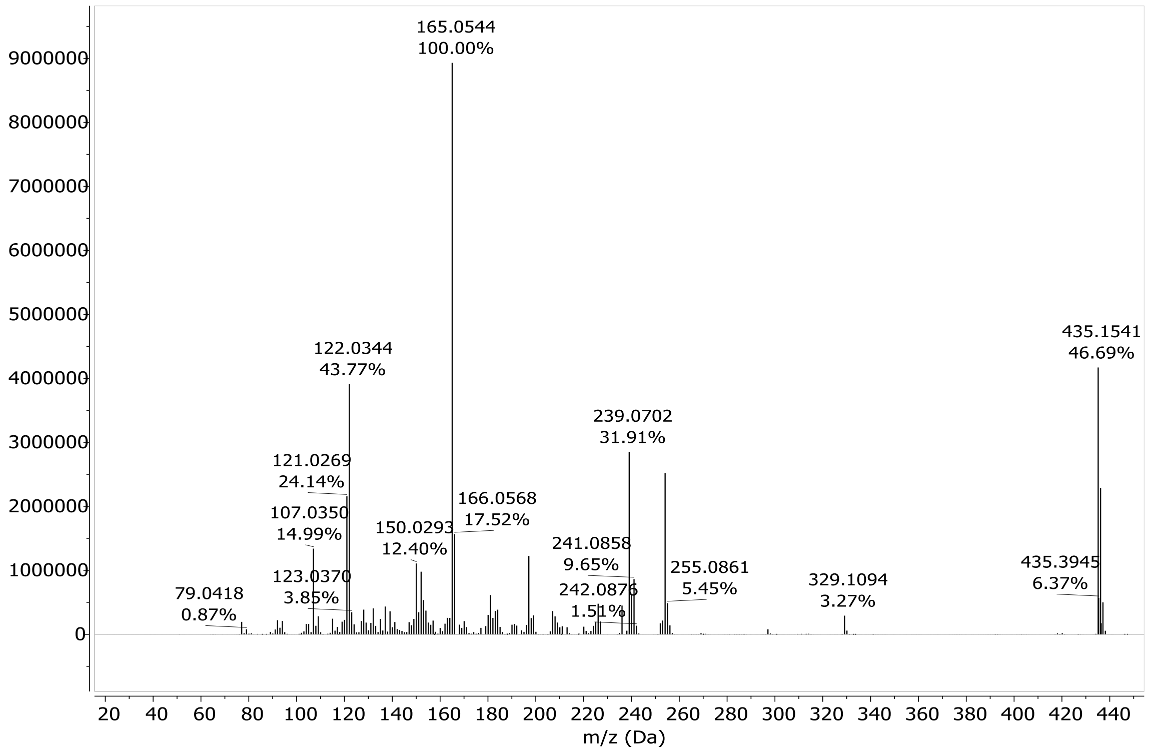
165.0544
100.00%
435.1541
46.69%
122.0344
43.77%
239.0702
31.91%
121.0269
24.14%
166.0568
17.52%
107.0350
14.99%
150.0293
12.40%
241.0858
9.65%
255.0861
5.45%
435.3945
6.37%
123.0370
3.85%
329.1094
3.27%
79.0418
0.87%
242.0876
1.51%
m/z (Da)

N'-((*E*)-2,4-dihydroxy-6-((*E*)-4-hydroxystyryl)benzylidene)-3,4-dimethoxybenzohydrazide (**6g**)

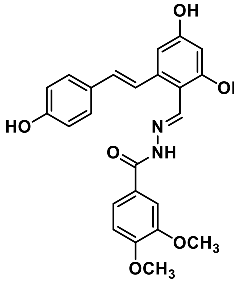

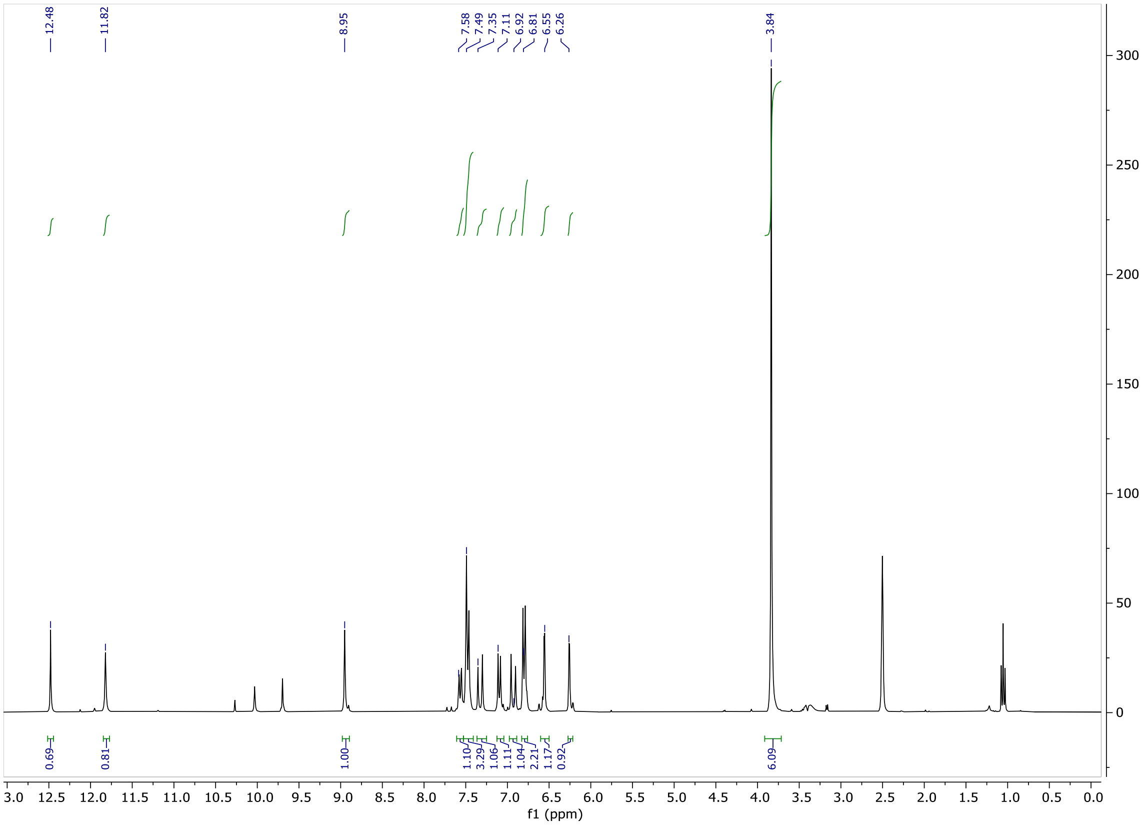

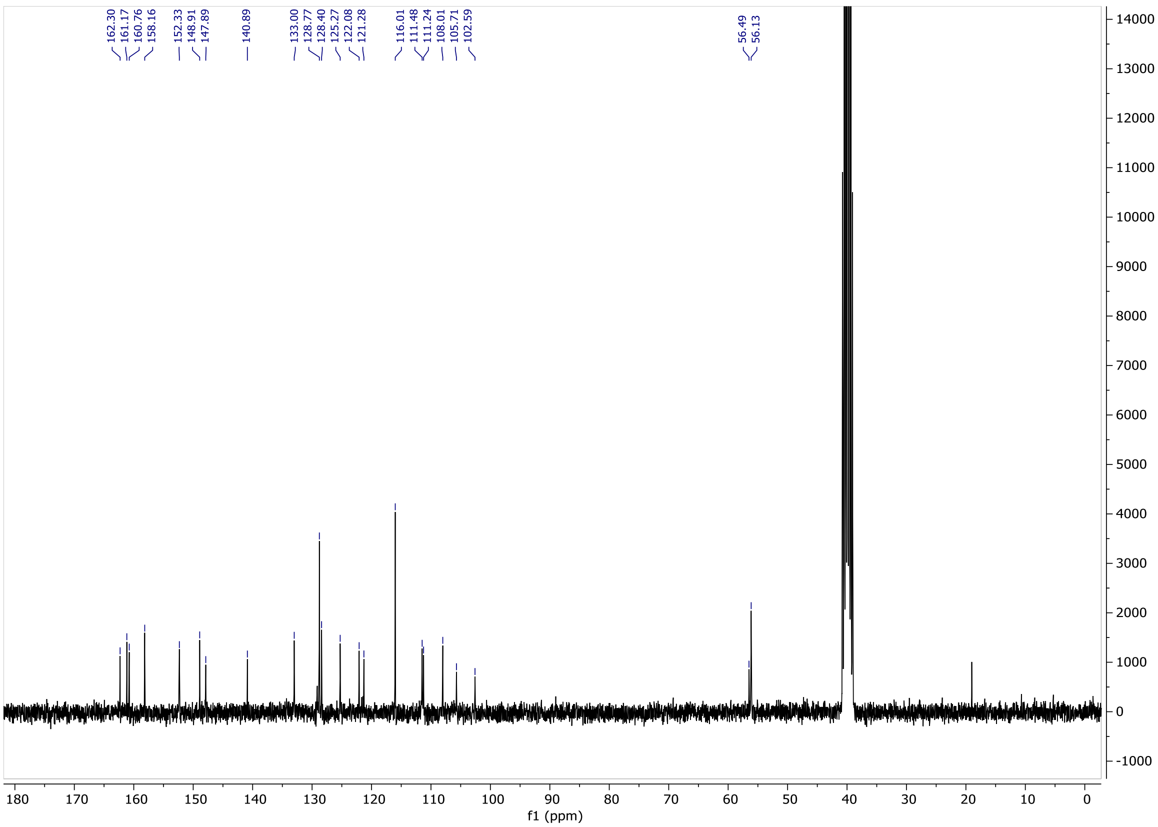
162.30
161.17
160.76
158.16
152.33
148.91
147.89
140.89
133.00
128.77
128.40
125.27
122.08
121.28
116.01
111.48
111.24
108.01
105.71
102.59
56.49
56.13
f1 (ppm)

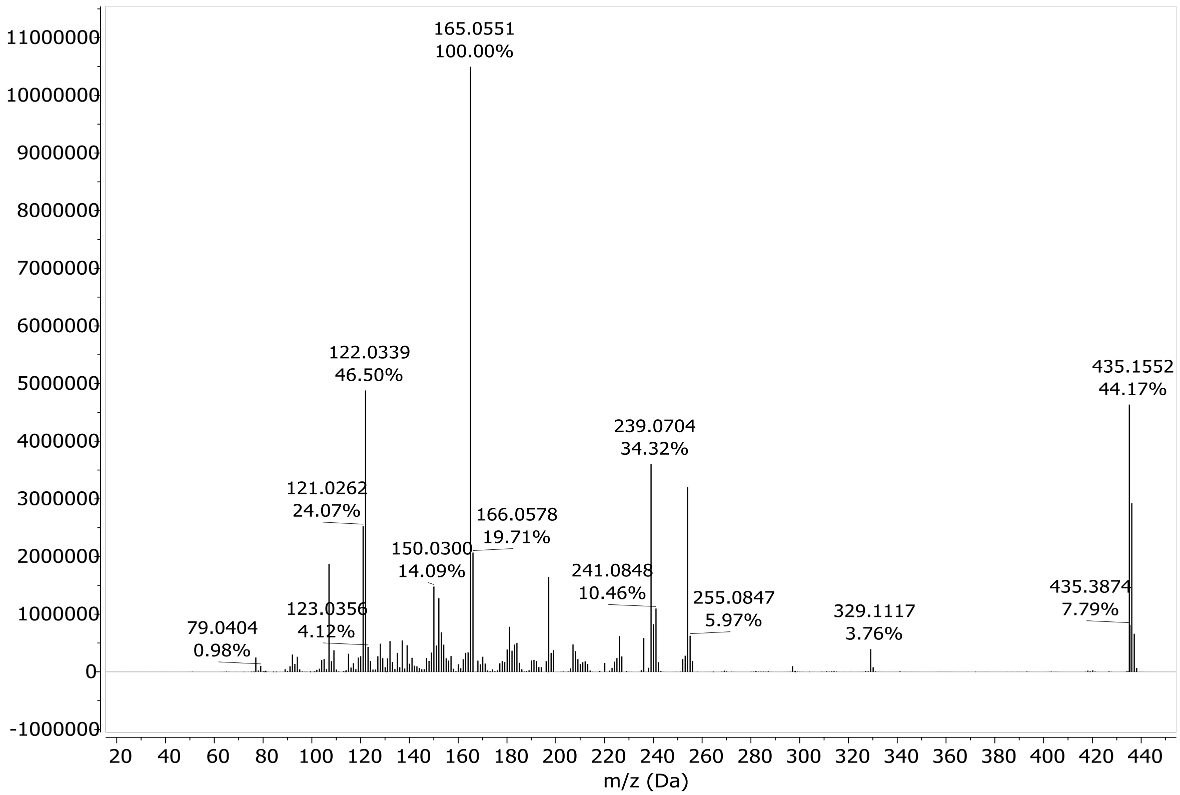
165.0551
100.00%
122.0339
46.50%
435.1552
44.17%
239.0704
34.32%
121.0262
24.07%
166.0578
19.71%
150.0300
14.09%
241.0848
10.46%
255.0847
5.97%
123.0356
4.12%
79.0404
0.98%
329.1117
3.76%
435.3874
7.79%
m/z (Da)

N'-((*E*)-2,4-dihydroxy-6-((*E*)-4-hydroxystyryl)benzylidene)-3,5-dimethoxybenzohydrazide (**6h**)

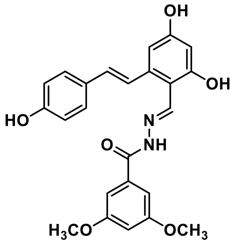

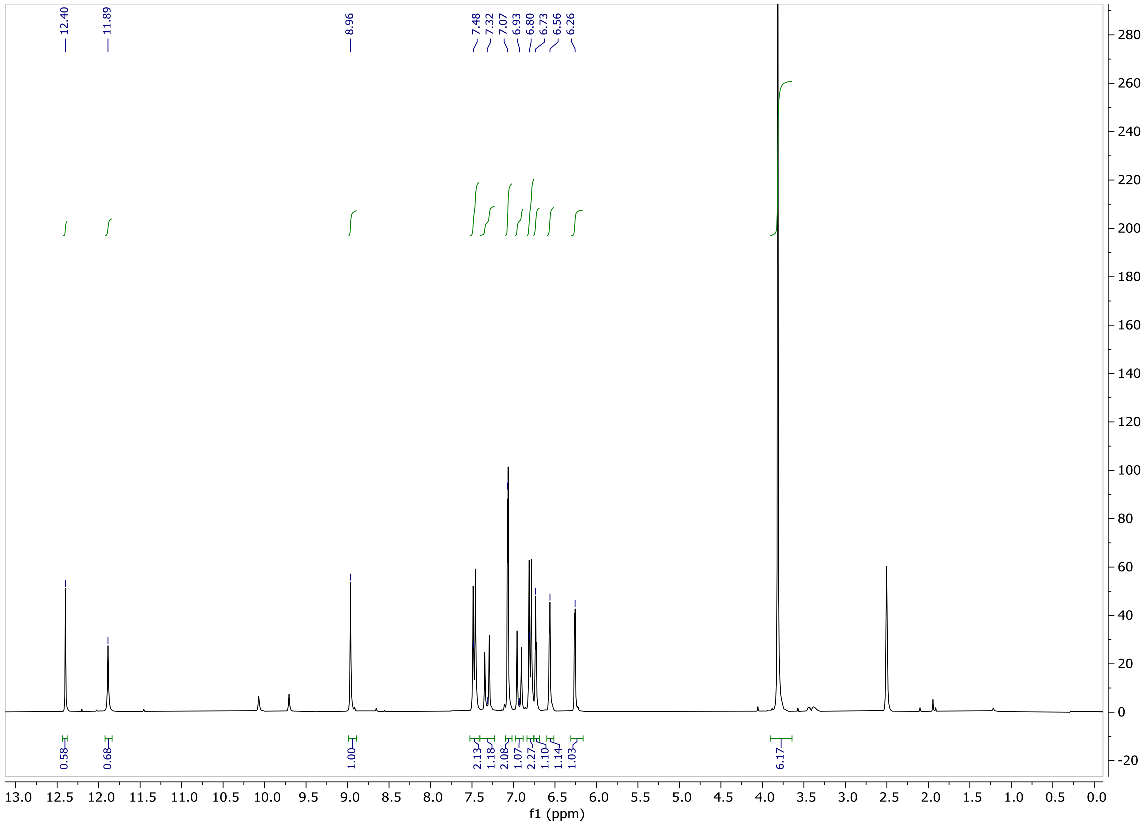

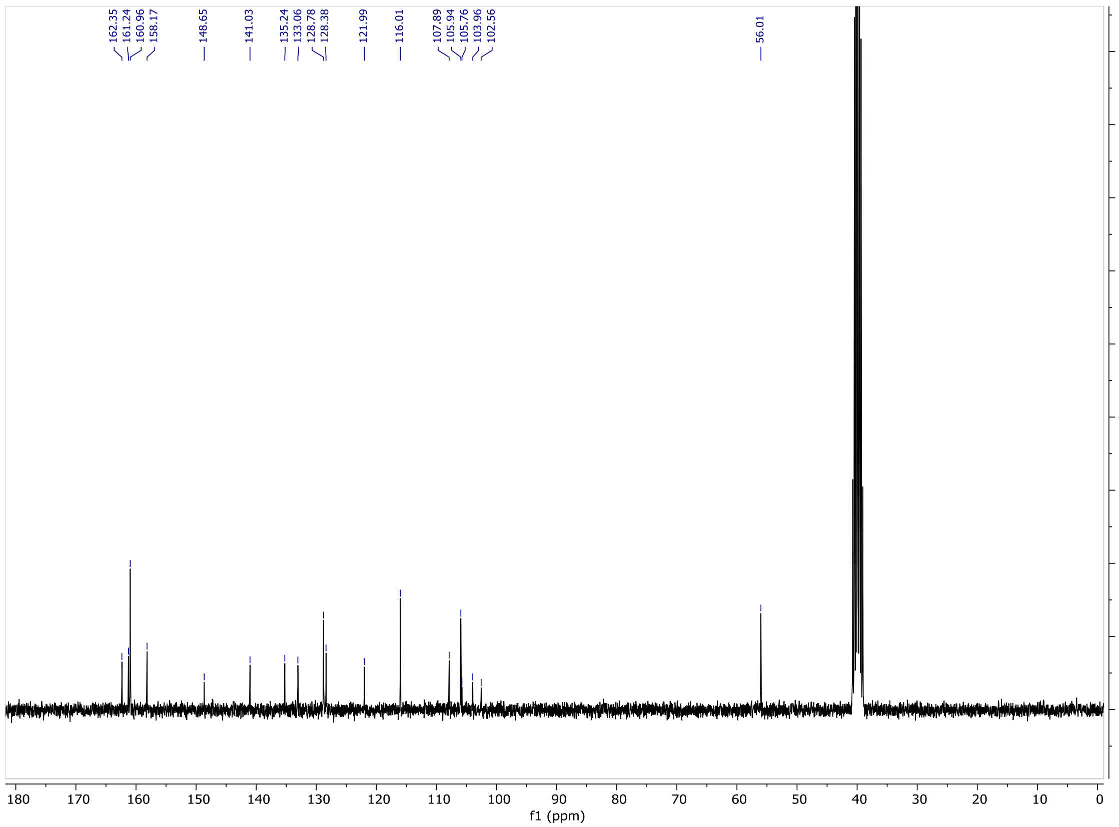

162.35
161.24
160.96
158.17
148.65
141.03
135.24
133.06
128.78
128.38
121.99
116.01
107.89
105.94
105.76
103.96
102.56
56.01
f1 (ppm)

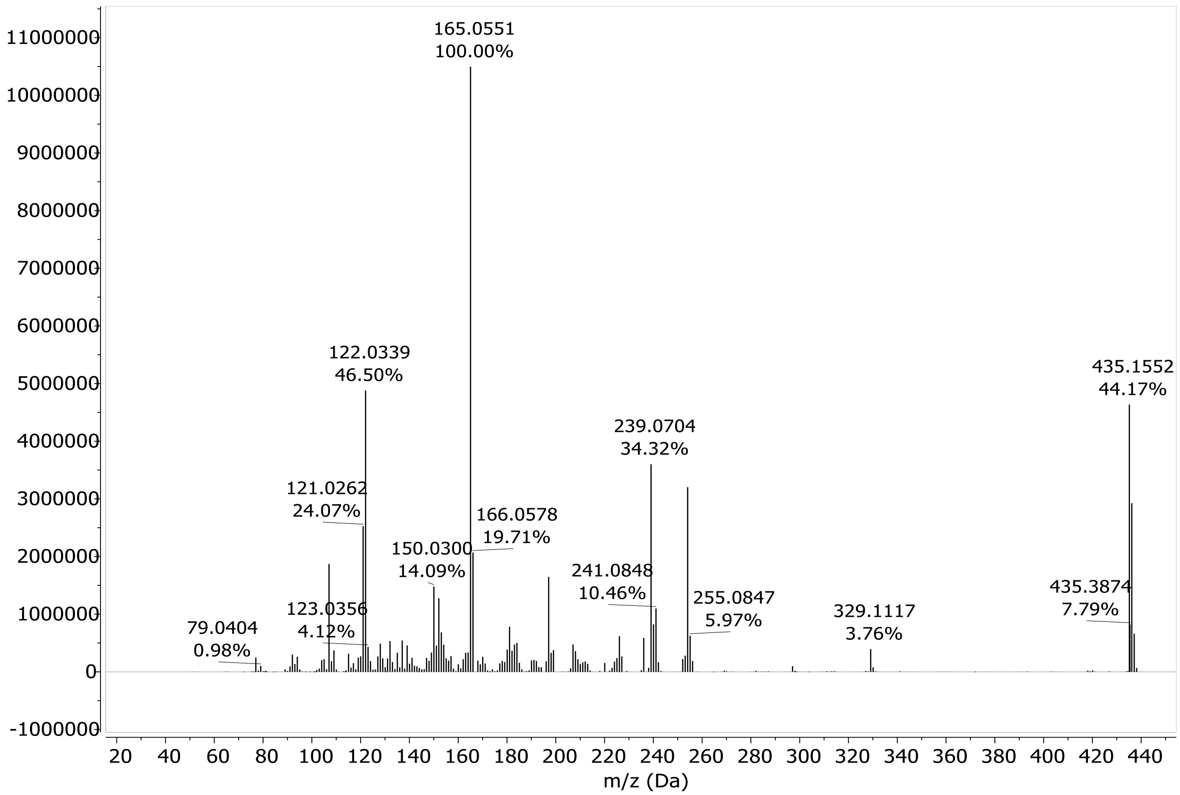

165.0551
100.00%
122.0339
46.50%
435.1552
44.17%
239.0704
34.32%
121.0262
24.07%
166.0578
19.71%
150.0300
14.09%
241.0848
10.46%
255.0847
5.97%
435.3874
7.79%
123.0356
4.12%
79.0404
0.98%
329.1117
3.76%
m/z (Da)

N′-((*E*)-2,4-dihydroxy-6-((*E*)-4-hydroxystyryl)benzylidene)isonicotinohydrazide (**7**)

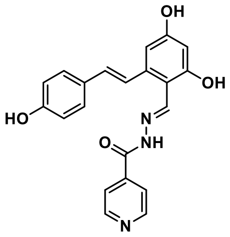

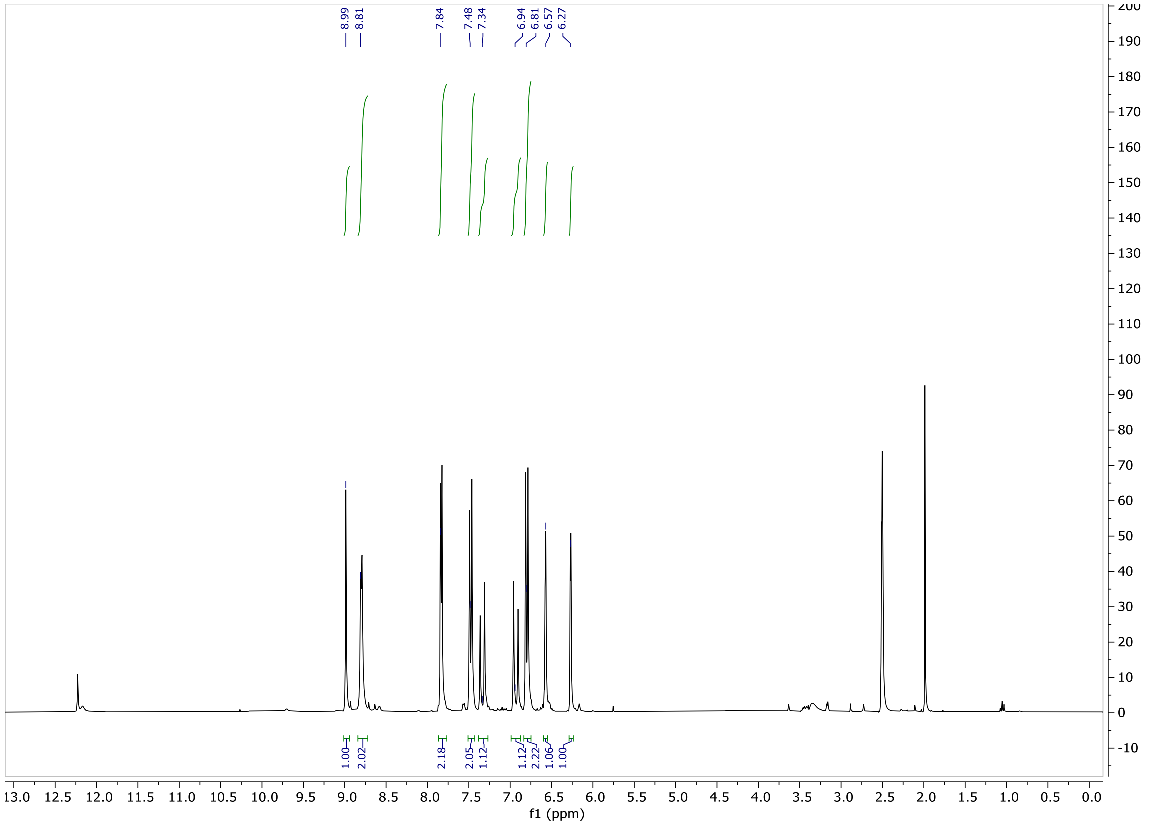

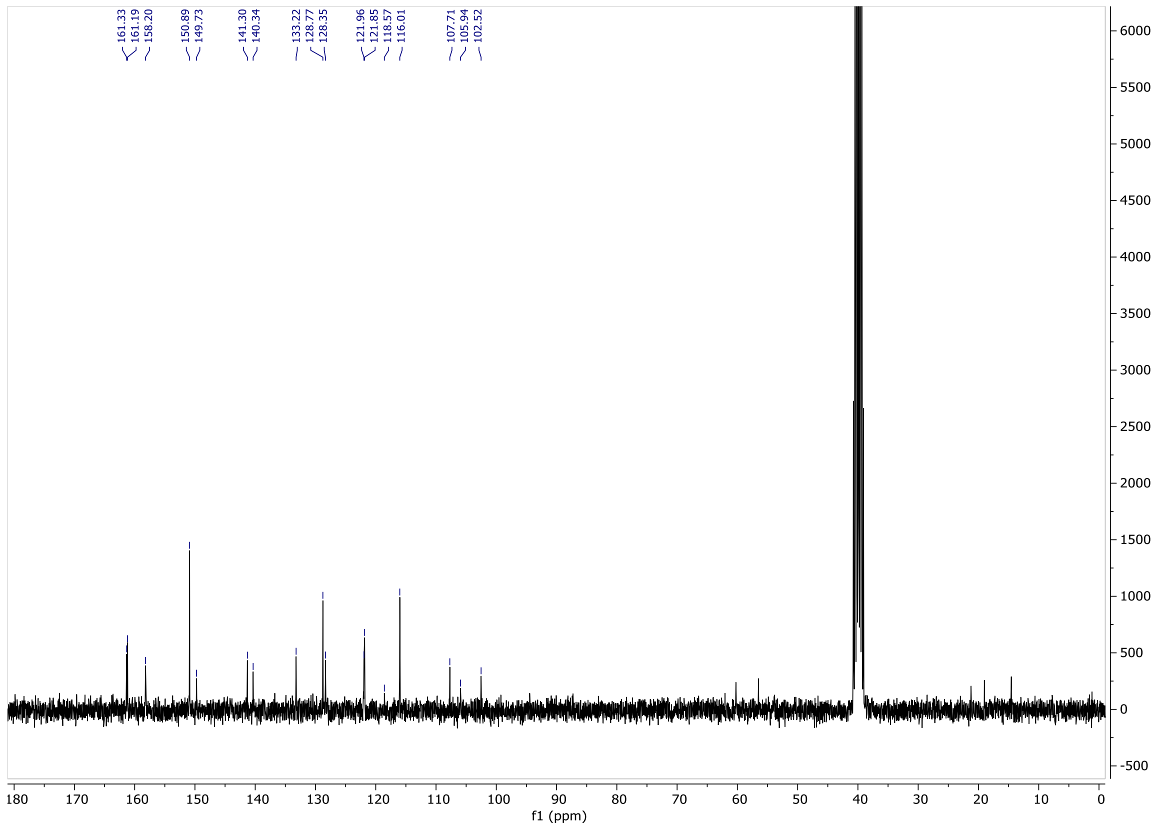
161.33
161.19
158.20
150.89
149.73
141.30
140.34
133.22
128.77
128.35
121.96
121.85
118.57
116.01
107.71
105.94
102.52
f1 (ppm)

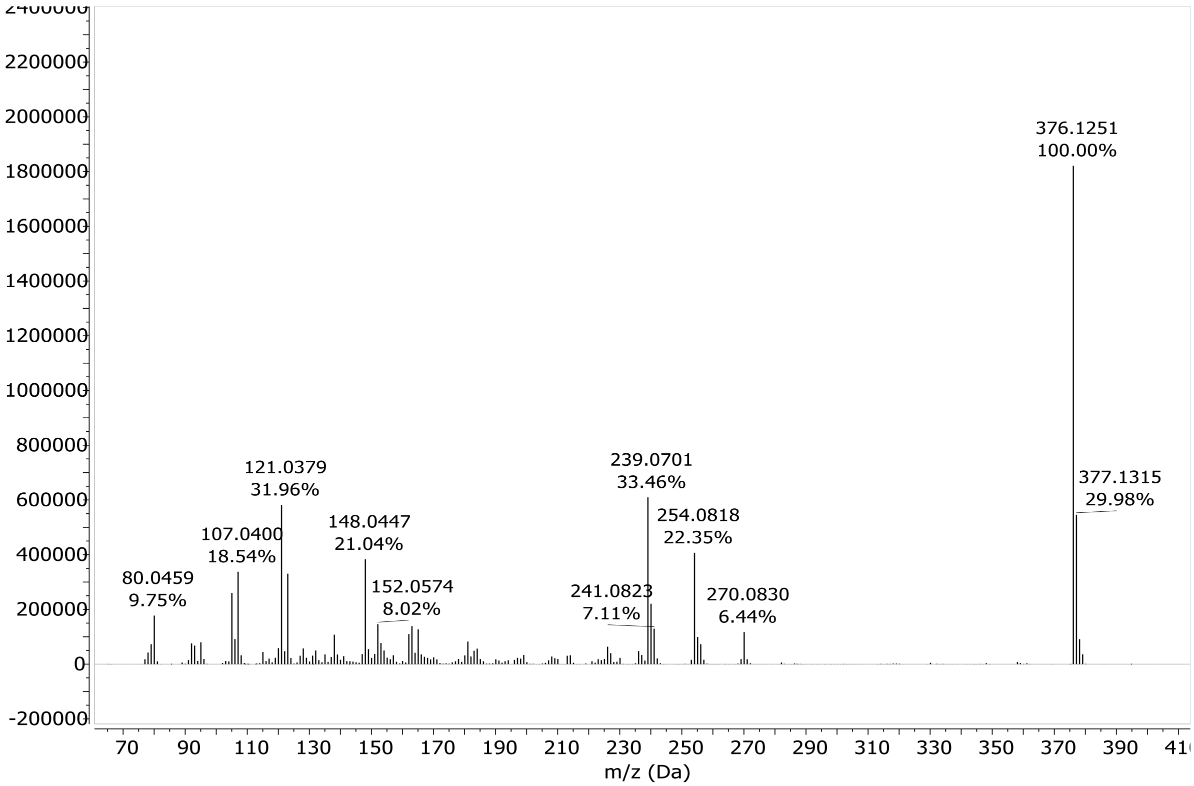
376.1251
100.00%
377.1315
29.98%
239.0701
33.46%
254.0818
22.35%
121.0379
31.96%
107.0400
18.54%
148.0447
21.04%
152.0574
8.02%
80.0459
9.75%
241.0823
7.11%
270.0830
6.44%
m/z (Da)

2-((E)-2,4-dihydroxy-6-((E)-4-hydroxystyryl)benzylidene)hydrazine-1-carbothioamide (**8**)

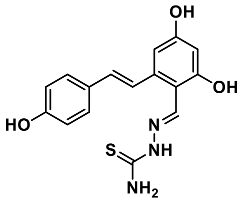

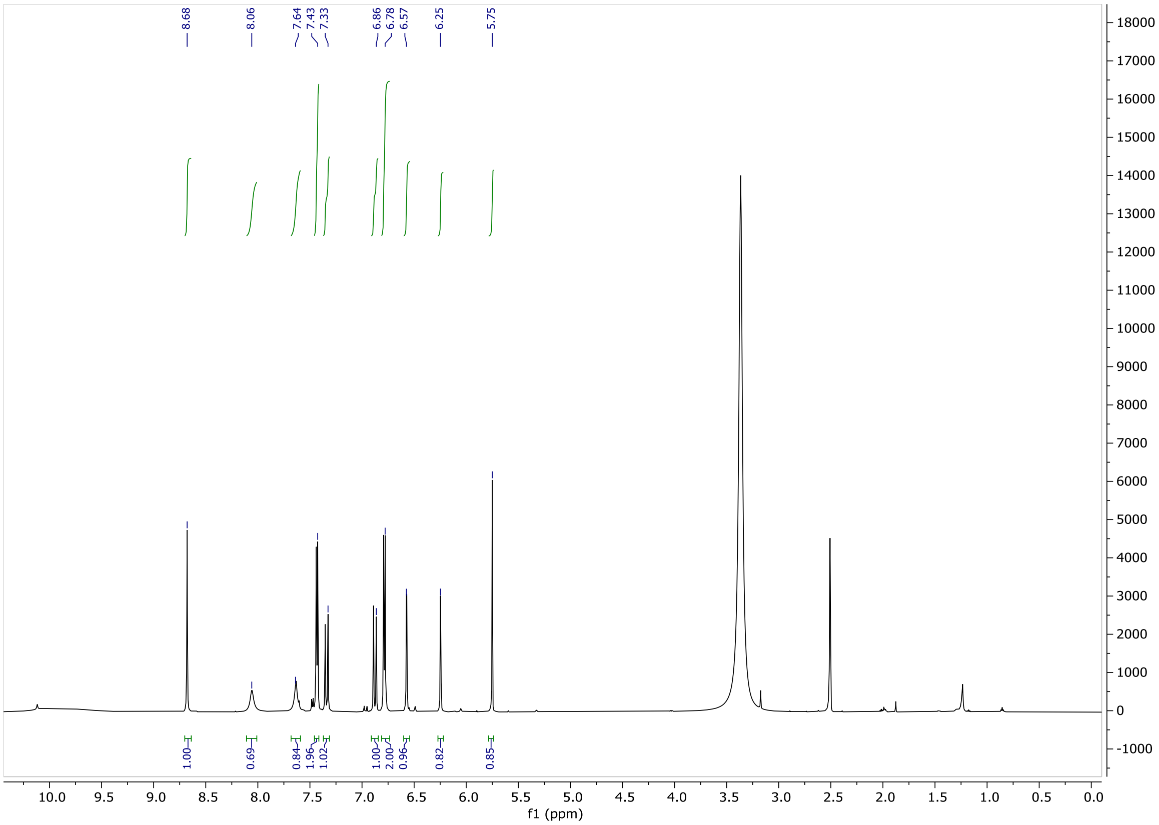

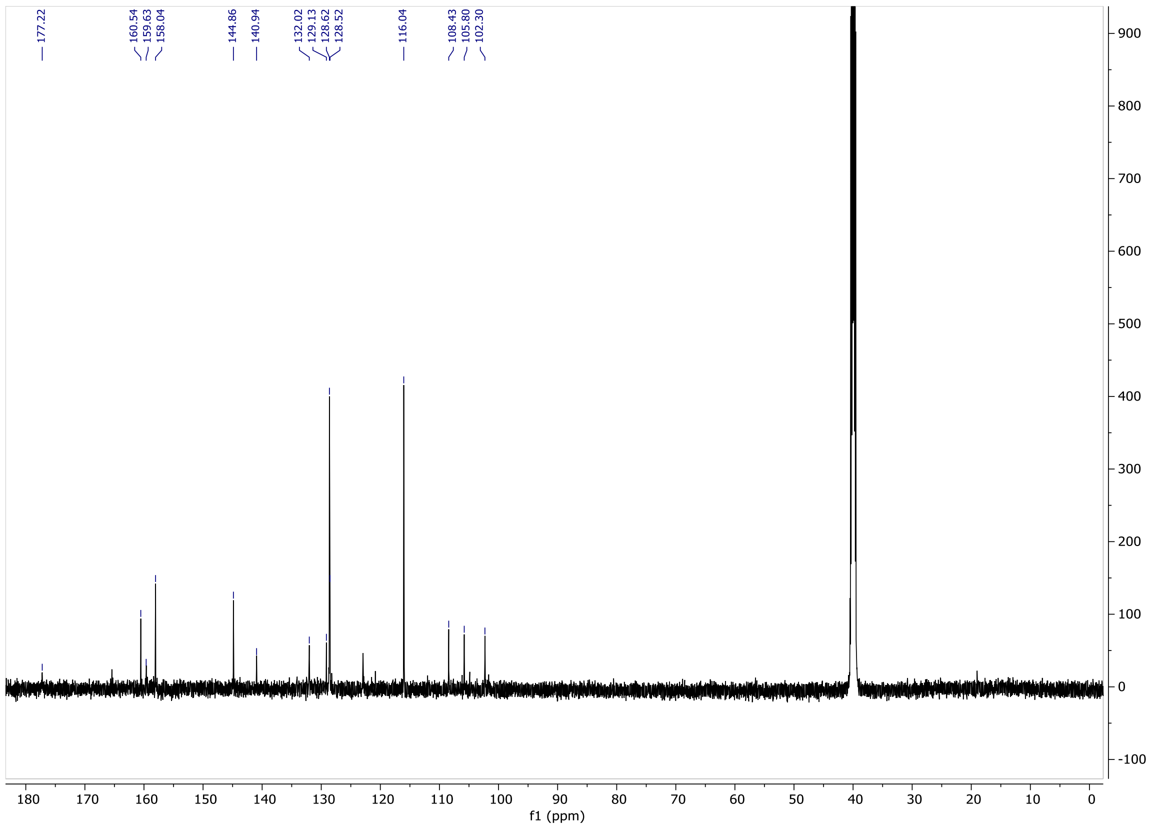

177.22
160.54
159.63
158.04
144.86
140.94
132.02
129.13
128.62
128.52
116.04
108.43
105.80
102.30
f1 (ppm)

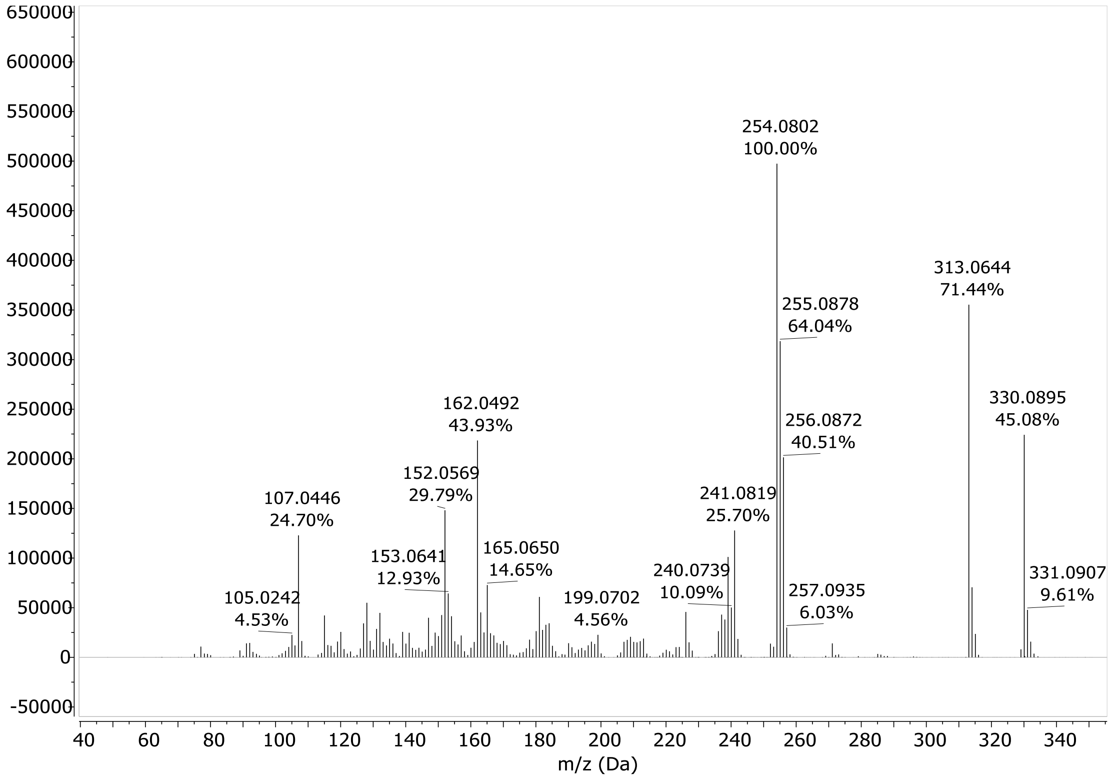

254.0802
100.00%
313.0644
71.44%
255.0878
64.04%
330.0895
45.08%
256.0872
40.51%
162.0492
43.93%
152.0569
29.79%
241.0819
25.70%
107.0446
24.70%
165.0650
14.65%
153.0641
12.93%
240.0739
10.09%
331.0907
9.61%
105.0242
4.53%
199.0702
4.56%
257.0935
6.03%
m/z (Da)

S2. HPLC analysis

Chromatogram and Results

Injection Details

Injection Name:	**7**	*Run Time (min):*	**20,00**
Vial Number:	**RB1**	*Injection Volume:*	**20,00**
Injection Type:	**Unknown**	*Channel:*	**UV_VIS_1**
Calibration Level:		*Wavelength:*	**359**
Instrument Method:	**Muestras R5 y S1**	*Bandwidth:*	**n.a.**
Processing Method:	**Basic Quantitative**	*Dilution Factor:*	**1,0000**
Injection Date/Time:	**08/sep./22 11:45**	*Sample Weight:*	**1,0000**

Chromatogram

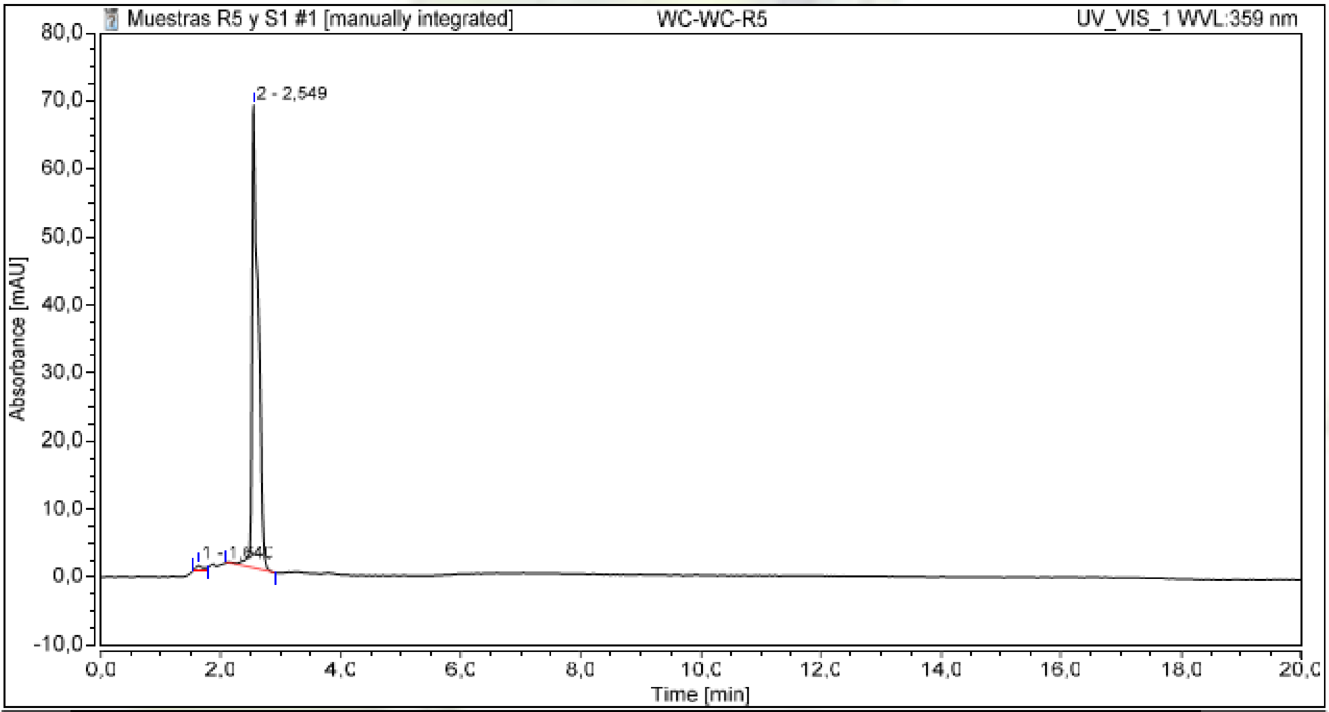

Integration Results

No.	Peak Name	Retention Time min	Area mAU*min	Height mAU	Relative Area %	Relative Height %	Amount n.a.
1		1,640	0,044	0,569	0,53	0,83	n.a.
2		2,549	8,156	68,097	99,47	99,17	n.a.
**Total:**			**8,200**	**68,666**	**100,00**	**100,00**	

Chromatogram and Results

Injection Details

Injection Name:	**6E**	*Run Time (min):*	**20,00**
Vial Number:	**RB2**	*Injection Volume:*	**20,00**
Injection Type:	**Unknown**	*Channel:*	**UV_VIS_1**
Calibration Level:		*Wavelength:*	**359**
Instrument Method:	**Muestras R5 y S1**	*Bandwidth:*	**n.a.**
Processing Method:	**Basic Quantitative**	*Dilution Factor:*	**1,0000**
Injection Date/Time:	**08/sep./22 12:06**	*Sample Weight:*	**1,0000**

Chromatogram

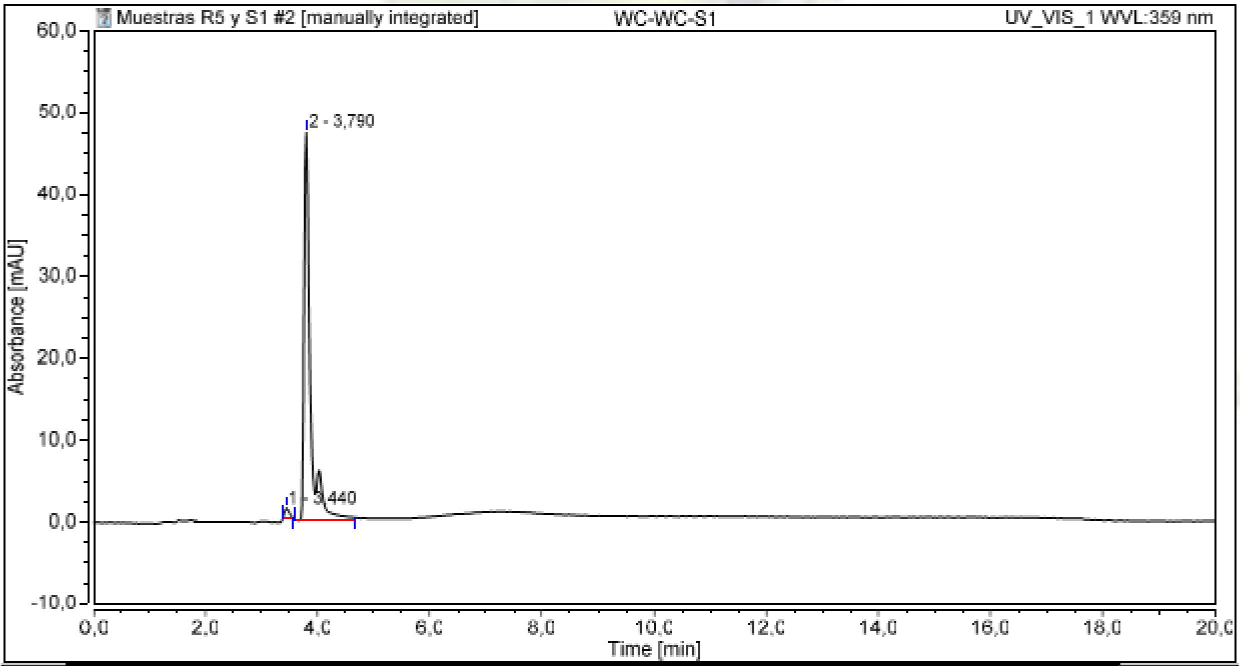

Integration Results

No.	Peak Name	Retention Time<br>min	Area<br>mAU*min	Height<br>mAU	Relative Area<br>%	Relative Height<br>%	Amount<br>n.a.
1		3,440	0,111	1,180	1,85	2,44	n.a.
2		3,790	5,875	47,225	98,15	97,56	n.a.
**Total:**			**5,985**	**48,405**	**100,00**	**100,00**	
